# Computational and Experimental Study of Metal–Organic Frameworks (MOFs) as Antimicrobial Agents against *Neisseria gonorrhoeae*

**DOI:** 10.1021/acsami.4c15851

**Published:** 2025-03-27

**Authors:** Ravi Kant, Megha Prajapati, Pradip Das, Antonios G. Kanaras, Daman Saluja, Myron Christodoulides, Chhaya Ravi Kant

**Affiliations:** † Medical Biotechnology Laboratory, Dr. B. R. Ambedkar Center for Biomedical Research, University of Delhi, Delhi 110007, India; ‡ Molecular Microbiology, School of Clinical and Experimental Sciences, Faculty of Medicine, University of Southampton, Southampton SO16 6YD, U.K.; § Department of Applied Sciences and Humanities, Indira Gandhi Delhi Technical University for Women, Kashmiri Gate, Delhi 11006, India; ∥ Electronics Materials Lab, College of Science and Engineering, James Cook University, Townsville, QLD 4811, Australia; ⊥ School of Physics and Astronomy, University of Southampton, Southampton SO17 1BJ, U.K.

**Keywords:** Neisseria gonorrhoeae, gonorrhea, metal−organic frameworks, copper, antimicrobial, computational, material synthesis

## Abstract

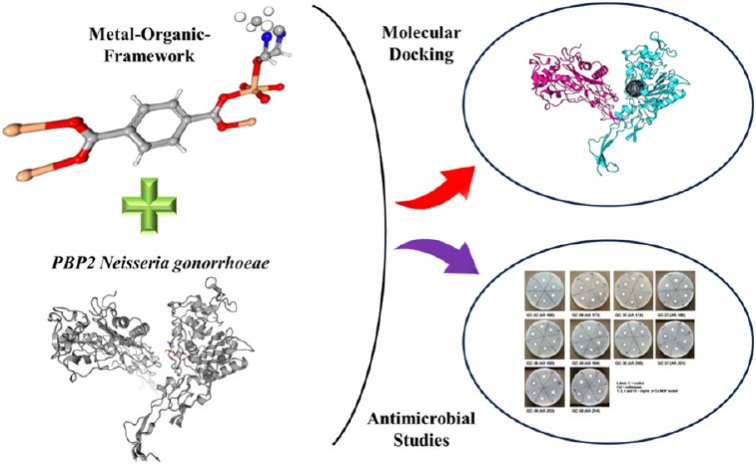

The emergence of drug-resistant superbugs poses a critical global health threat, necessitating innovative treatment strategies. *Neisseria gonorrhoeae* (Ng) causes a sexually transmitted disease called gonorrhea, and the bacterium has shown alarming resistance to conventional antibiotics, underscoring the urgent need for novel therapeutic approaches. In the current study, we interfaced computational biology and materials science to investigate the interactions between in-house synthesized metal–organic frameworks (MOFs) and the penicillin-binding protein 2 (PBP2) of Ng, a key target for β-lactam antibiotics. Using molecular docking and interaction analyses, we identified three promising MOFs, namely, Fe-BDC-258445, Cu-BDC-687690, and Ni-BDC-638866, with optimum binding scores and stable interactions. These scores indicated strong interactions with PBP2, suggesting their potential as therapeutic agents. Antimicrobial screening using a standard disk diffusion assay demonstrated that the Cu-BDC MOFs were bactericidal for multiple strains of Ng, whereas the Ni-BDC and Fe-BDC MOFs were nonbactericidal. The Cu-BDC MOF did not kill other Gram-negative bacteria, thus demonstrating specificity for Ng, and showed low toxicity for human Chang conjunctival epithelial cells *in vitro*. No significant leaching with biological activity was observed for the Cu-BDC MOF, and microscopy demonstrated the loss of gonococcal piliation and damage to the cell membrane. These findings underscore the potential of Cu-BDC MOFs as antimicrobial agents for further development.

## Introduction

Gram-negative bacterium *Neisseria gonorrhoeae* (Ng) is an obligate and predominantly sexually transmitted pathogen of humans that causes gonorrhea.^1^ Penicillin-binding protein 2 (PBP2) plays a role in the life cycle of the gonococcus and represents a potential target for inhibiting bacterial growth.^2^ PBP2 is a transpeptidase enzyme responsible for catalyzing the final step in forming the Gram-negative peptidoglycan layer by helping to cross-link the peptidoglycan chains, which provides structural integrity to the cell wall and helps to maintain shape and contain internal osmotic pressure.^3,4^ PBP2 is targeted by penicillin and other β-lactam antibiotics that function by inhibiting PBP2 transpeptidase enzyme activity.^5^ These antibiotics have a structure similar to that of the d-alanyl-d-alanine portion of peptidoglycan precursors. PBP2 binds to antibiotic molecules instead of peptidoglycan precursors, and consequently, the structural integrity of the bacterial cell wall is weakened or cross-linked, making it more susceptible to osmotic pressure and ultimately leading to cell lysis.^6,7^ In the past, a single dose of penicillin was sufficient to clear gonorrhea, but penicillin-resistant strains have emerged because of mutations in PBP2.^8^ In addition, some strains of *N. gonorrhoeae* have developed resistance to penicillin and other β-lactam antibiotics by acquiring altered forms of PBP2 through genetic mutations, horizontal gene transfer, and mechanisms such as transformation.^9^

*N. gonorrhoeae* produces four PBPs, namely, two high-molecular-weight trans-peptidases (class A PBP1 and class B PBP2) and two low-molecular-weight class C proteins (PBP3 and PBP4). The class C enzymes can be deleted with little effect on cell morphology and growth,^8,10^ whereas PBP1 and PBP2 are vital for cell viability and are targets for antibiotics. Indeed, PBP2 is considered the primary target of penicillin since it requires tenfold lower concentrations of penicillin for inhibition, compared with PBP1.^4^ Both PBP1 and PBP2 are prone to mutations, leading to penicillin-resistant gonococcal strains. For example, it is hypothesized that mutations in PBP2 can alter the structure of the active site, thereby lowering the acylation rate by penicillin, but this hypothesis is still subject to further validation.^8,10^ Such mutational trends have reinforced the need to develop new treatment regimens and antibacterial options for managing resistant gonococcal infections^2,11^ and underscore the importance of antibiotic stewardship.^6^

Recent research has highlighted several ligand–metal complexes as potential novel therapeutic agents for inhibiting the growth of *N. gonorrhoeae*.^7,12^ Also, hybrid organic–inorganic materials have emerged as promising antibacterial agents that function *via* the release of metal ions.^13^ Transition metals such as copper, iron, and nickel were shown to form strong bonds with active sites of proteins by electron transfer and enhance the affinity energy of the resulting complexes.^14,15^ In this regard, metal–organic frameworks (MOFs) have recently gained much attention. MOFs are composed of interconnected metal ions and organic linkers, forming bridges through coordinate bonds.^16−19^ MOFs, due to their high specific surface area and structural and synthetic adaptability, have been explored and utilized extensively in a diverse array of applications, encompassing gas storage, organocatalysis, photocatalysis, sensing, adsorption and separation, contaminant removal from gases or water, and electrochemical energy.^20−26^ Their applications also extended to biological and medical fields, offering key attributes such as (i) biodegradability through gradual metal-ion release forming metal complexes; (ii) high specific surface areas for enhanced drug encapsulation; (iii) low toxicity due to relatively labile metal–organic linker bonds; (iv) easy dispersal; (v) amenable functionalization; and (vi) unique properties like redox activity, variable coordination modes, and increased reactivity toward organic entities. Due to their high reactivity, MOFs strongly bond with proteins under normal conditions and form metal complexes that can potentially target and treat pathological disorders, including cancer, cardiovascular diseases, and microbial infections.^27−29^

Over the last decade, MOF-based materials have emerged as potential antibacterial agents.^30^ MOFs can target a wide range of bacteria, release metal ions in a controlled manner, and remain active for a long time.^31,32^ Bactericidal metal ions, including Fe^2+^, Ni^2+^, Ag^+^, Cu^2+^, Zn^2+^, Mn^2+^, and Co^2+^, and select organic antimicrobials, like porphyrins and imidazole, can be used to fabricate MOFs.^13^ These components exhibit modifiable acid/base/water stability and respond to distinct stimuli, such as laser irradiation or varying pH levels. The substantial porosity and specific surface area intrinsic to MOFs facilitate the incorporation of diverse substances within their pores, while the abundance of active surface groups augments the immobilization of other compounds on their surface.^33,34^ Computational modeling is a key tool for predicting molecular binding sites on proteins, while molecular docking (MD) specifically identifies the optimal binding sites and molecular arrangements. The effectiveness of these interactions is measured by the free energy associated with them, where a lower free energy means more stable interactions. MD can be used to interrogate molecular interactions with MOF structures and is useful for the virtual screening of potential molecules without having to synthesize them all.^11^ MOFs have been explored for various applications using MD, such as inhibiting SARS-CoV-2, delivering drugs like amoxicillin and ibuprofen, and treating wastewater.^35^ In the current study, we used the protein–ligand binding method of MD to investigate the binding interactions of MOFs with Ng-PBP2 and then tested the hypothesis that selected in-house synthesized MOFs were bactericidal toward gonococci.

## Experimental Methods

### Computational Analyses

#### Retrieval of Protein Structure and MOF Data Set

The structures of 19 potential MOFs were selected and retrieved from the Cambridge Crystallographic Data Centre (CCDC) MOF database (https://www.ccdc.cam.ac.uk/free-products/csd-mof-collection/). Their reported biological and physiochemical properties were studied before further analysis. The structure of *N. gonorrhoeae* penicillin-binding protein 2 (Ng-PBP2) (PDB: 3EQU) was obtained from the Protein Data Bank (PDB).^8^ To prepare the protein for analysis, the macromolecule file was modified using AutoDock Tools 1.5.6.^36^ The initial structure of the 3EQU protein consisted of two identical chains (A and B), each with 542 amino acid residues. Water molecules associated with both chains were removed. Additionally, polar hydrogens were added, and Kollman charges were assigned to the protein. Energy minimization was then performed in AutoDock Tools 1.5.6 to prepare the target protein for MD and interaction analyses.

#### Identification of the Active Site

The active site for MOF binding on the surface of Ng-PBP2 was predicted using DeepFold,^37^ which is a deep learning-based server that employs spatial restraint-guided structure prediction. Given the role of Ng-PBP2 in cell wall biosynthesis, we targeted residues that were typically involved in peptidoglycan synthesis and cross-linking. Both the A and B chains of Ng-PBP2 were analyzed using DeepFold. The conserved nature of these active site residues instilled confidence in the relative accuracy of our predicted binding site, reinforcing the validity of the adopted protein structure for subsequent analyses. Furthermore, to validate our findings, we cross referenced the identified active sites with the previously reported literature.^2,8^ Additionally, we confirmed the reliability of our identified active site predictions by repeating targeted docking experiments and creating the binding cavity by using the same set of residues.

#### Molecular Docking (MD)

MD studies were performed using AutoDock Vina,^36^ which allowed us to understand the spatial arrangement of interacting atoms and residues. This enabled us to assess the binding affinities of the MOFs and explore the precise conformations by which these MOFs fit into the protein’s surface binding region, which is useful for predicting the binding affinity of test MOF compounds against Ng-PBP2.^11^ The identified active site of the Ng-PBP2 protein was enclosed in a docking grid box defined with *x*, *y*, and *z* centers set at 13.192, −15.534, and 31.999, respectively; the grid dimensions were 40 Å × 40 Å × 40 Å, with a spacing of 0.375 Å. MOF structures were prepared using AutoDock Tools and saved as pdbqt files. Schematic depictions of the ligand–receptor binding site interactions were generated using AutoDock and the PLIP web server.^38^

### Synthesis and Characterization of MOFs

Fe(NO_3_)_3_·9H_2_O, Ni(NO_3_)_2_·6H_2_O, 2-hydroxyterephthalic acid (H_2_-BDC), dimethylformamide (DMF), pyridine, hydrogen peroxide (H_2_O_2_), Cu(NO_3_)_2_·6H_2_O, sodium hydroxide (NaOH), and deionized (DI) water were procured from distributors for Merck, India. All reagents were of analytical grade and used without further purification.

#### Synthesis of Fe-BDC MOFs

In a typical synthesis, a mixture of Fe(NO_3_)_3_·9H_2_O (0.808 g), H_2_BDC (0.340 g), DMF (20 mL), pyridine (1 mL), and H_2_O_2_ (0.5 mL) was transferred into a 50 mL Teflon-lined autoclave reactor. The solution was maintained at 180 °C for 48 h. After the reaction, the dark brown solution was centrifuged, and the sediment was washed multiple times with DMF. The final product was dried at 60 °C overnight to obtain Fe-BDC MOFs.^39^

#### Synthesis of Ni-BDC MOFs

A mixture of Ni(NO_3_)_2_·6H_2_O (0.808 g) and H_2_BDC (0.340 g) was dissolved in 45 mL of deionized water. NaOH was added to the mixture until the pH reached 8. The solution was then poured into a 50 mL Teflon-lined stainless steel autoclave and heated at 150 °C for 72 h. The final product was collected by filtration, washed multiple times with DI water, and dried at ambient temperature for 8 h to yield Ni-BDC MOFs.^40^

#### Synthesis of Cu-BDC MOFs

Cu(NO_3_)_2_·6H_2_O (0.0080 mol) and H_2-_BDC (0.0055 mol) were mixed in 50 mL of DMF. The mixture was transferred to a stainless steel autoclave and heated at 100 °C for 5 h. The final product was collected by filtration, washed multiple times with DI water, and dried at ambient temperature for 8 h to obtain Cu-BDC MOFs.^41^

#### Characterization of Synthesized MOFs

Fourier transform infrared (FTIR, Shimadzu, Japan) spectra were collected to obtain information about chemical bonding vibrations and functional groups of the material. FTIR analysis was used to investigate the molecular vibrations present in the prepared samples, identifying various functional groups in the Ni-BDC, Cu-BDC, and Fe-BDC MOFs. Scanning electron microscopy (SEM) was used to analyze the surface morphology and structural integrity of the synthesized MOFs. The surface morphology, elemental analysis, and energy-dispersive spectroscopy (EDS) of the samples were examined using a JEOL NeoScope SEM instrument to provide insights into the size, shape, and distribution of the particles. The specific surface area and porosity of the material were analyzed using the Brunauer–Emmett–Teller (BET) method.^42^ Measurements were conducted with a Nova Touch Quantachrome LX2 instrument under a nitrogen (N_2_) flow. Prior to the analysis, the samples were degassed at 200 °C for 6 h to ensure accurate results. X-ray diffraction (XRD) analysis was performed by using a Bruker D8 Advance diffractometer equipped with Cu Kα radiation. The thermal stability of samples was analyzed using a Model TGA HiRes1000, operating from RT to 1100°C, top loading, under a N_2_ environment.

### Bacterial Strains and Antimicrobial Testing

#### Bacterial Strains

*N. gonorrhoeae* strain P9-17, a 1B-26 serovar isolate (ND: P1.18-10,43: F1-26: ST-1926, Pil^+^Opa_b_^+^), was originally isolated from a patient with gonococcal prostatitis.^43^ P9-17 is our reference laboratory strain, which we have used extensively in gonococcal pathogenesis studies and vaccine development. A panel of 50 *N. gonorrhoeae* isolates assembled by the Centers for Disease Control and Prevention (CDCP) in collaboration with the Food and Drug Administration (FDA) was also obtained (Antibiotic/Antimicrobial Resistance Isolate Bank, https://www.cdc.gov/drugresistance/resistance-bank/currently-available.html). Isolates showing the highest minimum inhibitory concentration (MIC) values for ceftriaxone (Merck, Gillingham, Dorset, U.K.) were selected for testing with the compounds. Gonococci were grown on supplemented GC agar plates^44^ incubated at 37 °C in an atmosphere containing 5% (v/v) CO_2_.

*Acinetobacter baumannii* ATCC19606 was obtained from LGC Standards, Teddington, U.K. *Pseudomonas aeruginosa* strain PAO1 (Holloway1C Stanier131) was obtained from the National Collection of Industrial, Food, and Marine Bacteria, U.K. *Klebsiella pneumoniae* NCTC 9634 was obtained from the National Collection of Type Cultures, Porton Down, Salisbury, U.K. *Escherichia coli* DSM (018:K1:H-) is a spontaneous nalidixic acid-resistant strain of *E. coli* RS228, which was originally isolated from a fecal specimen from a healthy individual.^45^ This strain was shown to be pathogenic in the infant rat model of bacteremia and meningitis.^46^ All of these Gram-negative bacteria were grown on nutrient agar plates at 37 °C in an atmosphere containing 5% (v/v) CO_2_.

For the agar diffusion assays, bacteria were suspended in Dulbecco’s modified phosphate-buffered saline (pH 7.4, PBSB), with turbidity adjusted to 0.5 McFarland equivalence turbidity standard (Remel, U.K.), which is ∼2 × 10^8^ colony forming units/mL. Aliquots of 50 μL were spread over the surface of GC agar and NA plates.

#### Agar Diffusion Assay

The standard agar diffusion protocol described by European Committee on Antimicrobial Susceptibility Testing (EUCAST, Version 12.0, January 2024, https://www.eucast.org/) was followed. Antibiotics ceftriaxone, ciprofloxacin, and polymyxin B and the Cu-BDC MOF were dissolved in sterile ultrahigh-quality (UHQ) water, whereas the Ni-BDC MOF and Fe-BDC MOF compounds were suspended in methanol (100%). The metal compounds were suspended by sonication in a water bath for 2–3 h at room temperature. Dilutions of the compounds and ceftriaxone were made in respective diluents, and 20 μL volumes were spotted onto 6 mm Whatman AA discs (Merck, U.K.). Discs were placed onto the surfaces of GC-inoculated agar plates and incubated overnight at 37 °C in an atmosphere containing 5% (v/v) CO_2_. Plates were inspected, and the inhibition diameter zones were measured.

#### Preparation of Solutions for Ion Leaching Experiments

The method for preparing leaching materials was based on Behzadinasab et al.,^47^ with some modifications, notably extended suspension time for the Cu-BDC MOF and testing of the unfiltered material. The Cu-BDC MOF (10 mg/mL) was suspended in UHQ and sonicated to dispersion in a water bath for 2.5 h, as described above. To measure the biological activity of any dissolved leachate content, suspended particles were removed by centrifugation at 4000 rpm for 4 min and then filtered using a 0.22 μM filter and syringe. Unfiltered leachate was also prepared. Leachate volumes were prepared in UHQ were matched to the equivalent concentrations of 10, 5, 3, and 1 mg/mL Cu-MOF, and aliquots of 20 μL were applied to 6 mm filter discs. Bactericidal killing was assessed using the agar disc diffusion assay, as described above, with ceftriaxone (1 μg/mL) and Cu-MOF (1–10 mg/mL) as positive controls. Negative controls were discs with UHQ alone.

#### Assessing the Cytotoxicity of Cu-BDC MOFs

Human Chang conjunctival epithelial cells (European Type Culture Collection, Porton Down, U.K.) were cultured in sterile 96-well cell culture plates (Nunc) at 37 °C in Dulbecco’s modified Eagle’s medium (DMEM) supplemented with Glutamax-1 and sodium pyruvate (Lonza, U.K.) and 10% (v/v) decomplemented fetal calf serum (dFCS) (Lonza). Cells were cultured in a humidified atmosphere at 37 °C with 5% (v/v) CO_2_. Prior to treatment with Cu-BDC MOFs, the medium was removed, the cells were washed to remove any dead cells, and fresh medium was added (180 μL/well). Next, 20 μL of test compound (twofold dilution of a 10 mg/mL prepared stock) was added per well in triplicate. Lysis solution (1% (w/v) sodium dodecyl sulfate in 0.1 M NaOH) was added as a positive control. Negative controls were cells alone without treatment. The plates were incubated for 18 h at 37 °C with 5% (v/v) CO_2_, and then 20 μL of resazurin (Merck, U.K.) was added to each well. The plate was incubated for further 4 and 18 h, and the absorbance was read at λ 570 and λ 595 nm for background correction on a SpectraMax iD3 plate reader. Cytotoxicity was calculated as the percentage inhibition of growth compared to the control well using the formula ((*O*_1_ × *A*_1_) – (*O*_1_ × *A*_2_)/(*O*_2_ × *P*_1_) – (*O*_1_ × *P*_2_)) × 100 where *O*_1_ is the molar extinction coefficient (*E*) of oxidized resazurin (blue) at 570 nm, *O*_2_ is the *E* of oxidized resazurin at 600 nm, *A*_1_ is the absorbance of test wells at 570 nm, *A*_2_ is the absorbance of test wells at 595 nm, *P*_1_ is the absorbance of the positive growth control well (cells plus resazurin but no test agent) at 570 nm, and *P*_2_ is the absorbance of the positive growth control well (cells plus resazurin but no test agent) at 595 nm.^48^

### Transmission Electron Microscopy (TEM)

*N. gonorrhoeae* P9-17 bacteria (5 × 10^5^ CFU/mL, 100 μL) in GC broth were mixed with 0.5 mg/mL (100 μL) and incubated in a 96-well plate overnight, in triplicate, in a humidified incubator at 37 °C with 5% (v/v) CO_2_. Controls consisted of untreated bacteria. After incubation, the volumes for each triplicate set were pooled, and the untreated and treated bacteria were collected by centrifugation (10,000 rpm for 5 min) and then washed with phosphate-buffered saline (PBS). The washed cells were fixed with 3% (v/v) glutaraldehyde and 4% (v/v) formaldehyde in piperazine-*N*,*N′*-bis(2-ethanesulfonic acid) (PIPES) buffer (0.1 M, pH 7.2) and washed again with Milli-Q water. The bacteria were suspended in Milli-Q water, and the bacterial suspensions were placed on Formvar/carbon-coated copper grids and then stained with 1% (w/v) ammonium molybdate in 0.1 M ammonium acetate buffer pH 7. The TEM images of the bacteria were acquired using a Hitachi HT7700 transmission electron microscope operating at 100 kV.

## Results

### Computational Analyses

#### Retrieval of Protein Structure and MOF Data Set

In this study, we started our analysis by retrieving the structures of 19 shortlisted MOFs from the publicly available MOF database. These MOFs were selected based on their promising biological and physiochemical properties, and an overview of their three-dimensional structures and characteristics such as molecular weight, surface area, pore size, and any previously reported biological activities and other known activities is provided in Table S1. The structure of Ng-PBP2 was retrieved from the PDB with accession code 3EQU. The Ng-PBP2 protein consists of two identical chains (A and B), each with 542 amino acid residues.

To prepare the Ng-PBP2 protein for MD and interaction analyses, we performed some molecular modifications using AutoDock Tools version 1.5.6. Initially, the structure encompassed both chains and coordinated water molecules, which were removed to eliminate potential interference during molecular docking. Polar hydrogens were added to the structure to accurately represent hydrogen-bonding interactions, and Kollman charges were integrated to account for electrostatic interactions within the protein.^49^ Furthermore, energy minimization was performed to ensure that the protein was in its most stable conformation, ready for the MD steps and interaction analyses.

#### Identification of the Active Site

**Figure 1:**
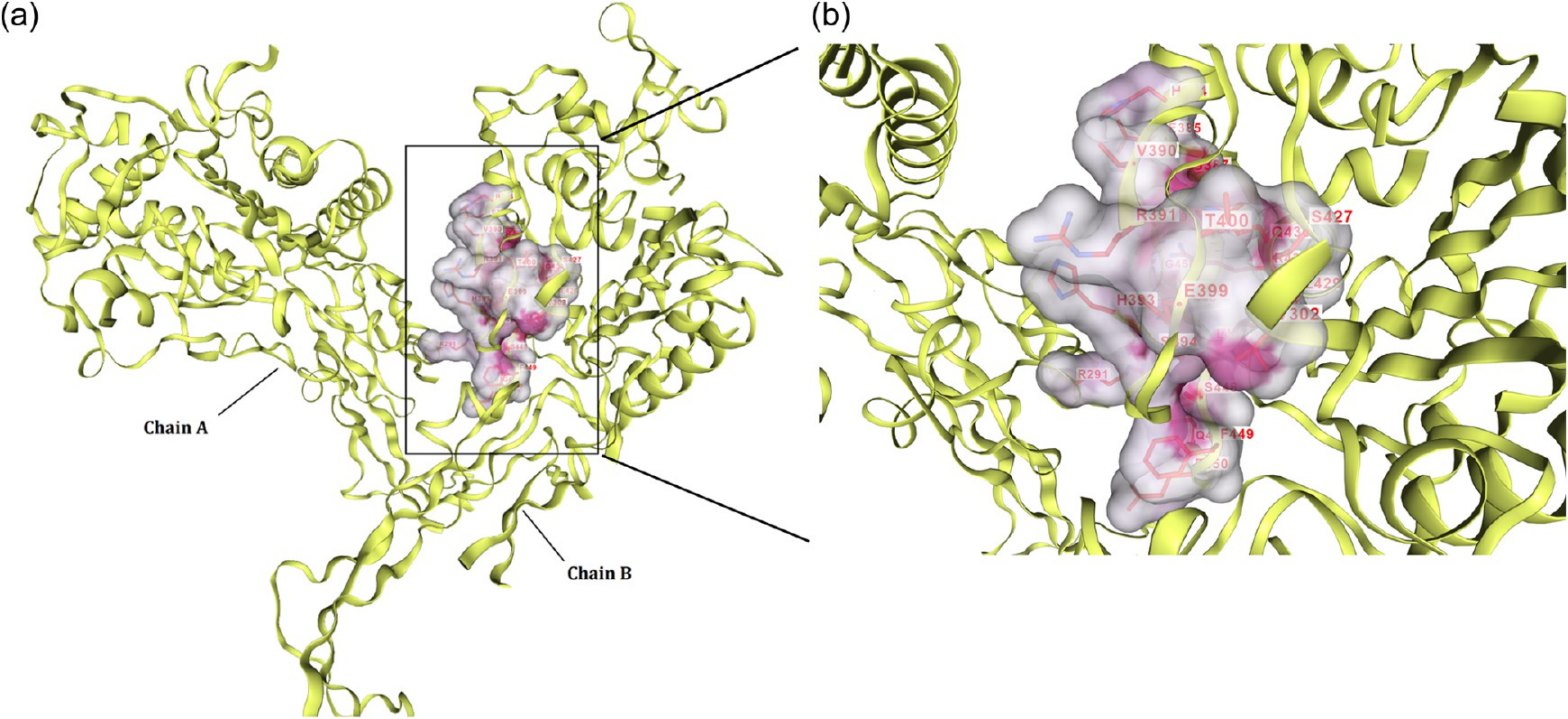
(a) Identified active site showing the binding cavity at the interface of two chains of Ng-PBP2. (b) Enlarged active site depicting the clear position and name of residues involved in the architecture of the active site.

DeepFold was used to analyze both A and B chains of the Ng-PBP2 protein to predict the active site for MOF binding to Ng-PBP2, and residues typically involved in peptidoglycan synthesis and cross-linking were targeted (Figure 1). In chain A, the following residues were identified as conserved and predicted to be part of the active site: Glu385, Gly387, Arg391, His393, Ser394, Phe396, Glu399, Leu429, Gln430, Arg433, Leu444, Pro446, Leu447, Gln457, and Lys459. In chain B, the following residues were identified as part of the predicted active site: Asn173, Tyr248, Asn252, Val255, Glu256, Tyr257, His258, Gln259, Ala260, Lys261, Ala262, Thr281, Pro282, Ala283, Tyr284, Asp285, Arg288, Pro289, Gly290, Arg291, Ala292, Asp293, and Gln296.

The conserved nature of these active site residues instilled confidence in the relative accuracy of our predicted binding site and reinforced the validity of the adopted protein structure for subsequent analyses. Furthermore, to validate our findings, we cross referenced the identified active sites with previously reported literature.^2,8^ We also confirmed our results and the reliability of our predicted active site by repeating targeted docking experiments and creating a binding cavity using the same set of residues.

#### MD and Interaction Analyses

**Figure 2:**
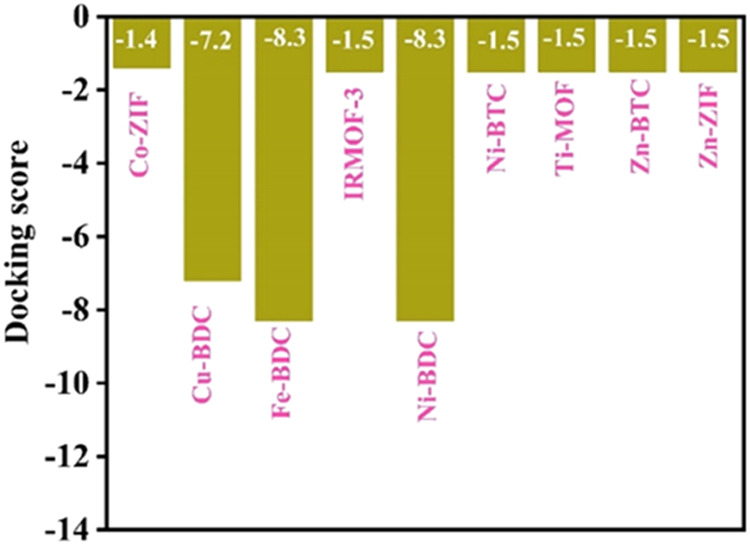
Comparative docking scores for MOFs with Ng-PBP2: docking scores of the top three MOFs—Cu-BDC-687690, Fe-BDC-258445, and Ni-BDC-638866—showing their binding affinities with Ng-PBP2. The figure highlights the relative scores, indicating that the three MOFs from the series of MOFs examined demonstrated the most favorable interactions with the protein.

On examining a panel of different MOFs (Co-ZIF, Cu-BDC, Fe-BDC, IRMOF-3, Ni-BDC, Ni-BTC, Ti-MOF, Zn-BTC, and Zn-Zif) and their MD scores, comparative analysis revealed three top-scoring MOFs, namely, Cu-BDC-687690, Fe-BDC-258445, and Ni-BDC-638866, which showed the most favorable docking interactions with Ng-PBP2 (Figure 2). These MOFs were shortlisted for further analysis, and a summary of the docking scores, interacting residues, and bond lengths in the metal complex conformations formed by these MOFs is given in Table 1, affirming their stable *in silico* interactions with Ng-PBP2.

**Table 1:** Summary of the Docking Scores, Contact Residues, and Bond Lengths (*A*_v_) with Metal Complexes for the Three Shortlisted MOFs

s. no.	ligand	docking score (kcal/mol)	contact residues	avg. bond length (metal complex)
1	Fe-BDC	–8.3	chain B: Ala73, Thr74, Arg75, Ser89, Leu165, Lys166, Arg167, Tyr169, Thr182, Asp183, Ile184, Asp185, Gly186, Leu200, Tyr201, Gly202, Pro286, Asn287, Pro289	2.3
2	Ni-BDC	–8.3	chain A: Glu385, Gly387, Arg391, His393, Ser394, Phe396, Glu399, Leu429, Gln430, Arg433, Leu444 Pro446, Leu447, Gln457, Lys459	2.15
			chain B: Asn173, Tyr248, Ala283, Tyr284, Asp285, Arg291, Asp293, Gln296	
3	Cu-BDC	–7.2	chain B: Arg167, Arg75, Ser89, Arg167, Thr182, Asn287, Ala73	2.26

**Figure 3:**
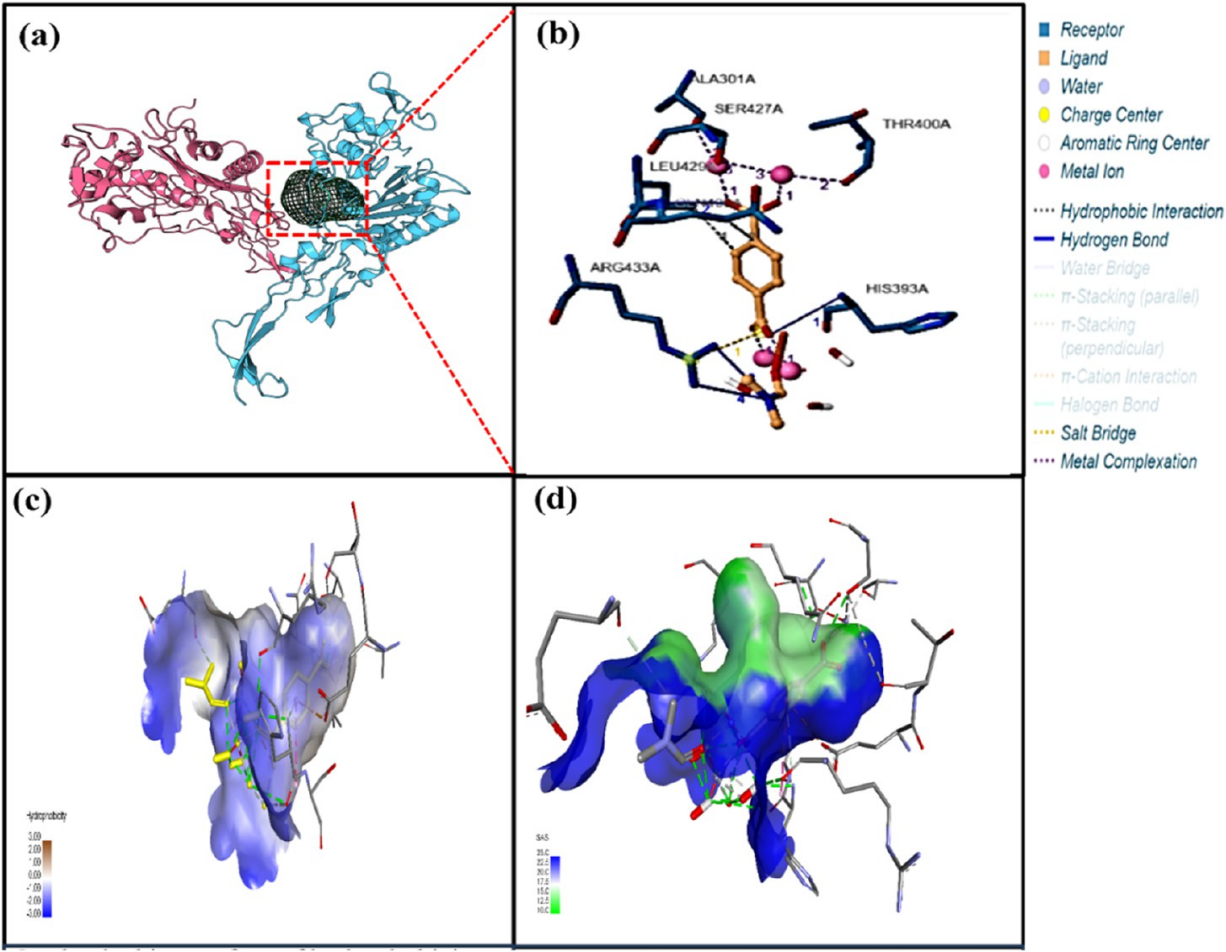
Binding interaction of Fe-BDC and the Ng-PBP2 protein. (a) MD of Fe-BDC with Ng-PBP2 represented with a ribbon conformation. (b) Crucial molecular interactions at the binding site. (c) Hydrophobicity of the protein–MOF docked complex. (d) Solvent accessibility surface diagram. Two different domains of bacterial protein are represented in red and blue, whereas the MOF is represented by the mesh (black).

**Figure 4:**
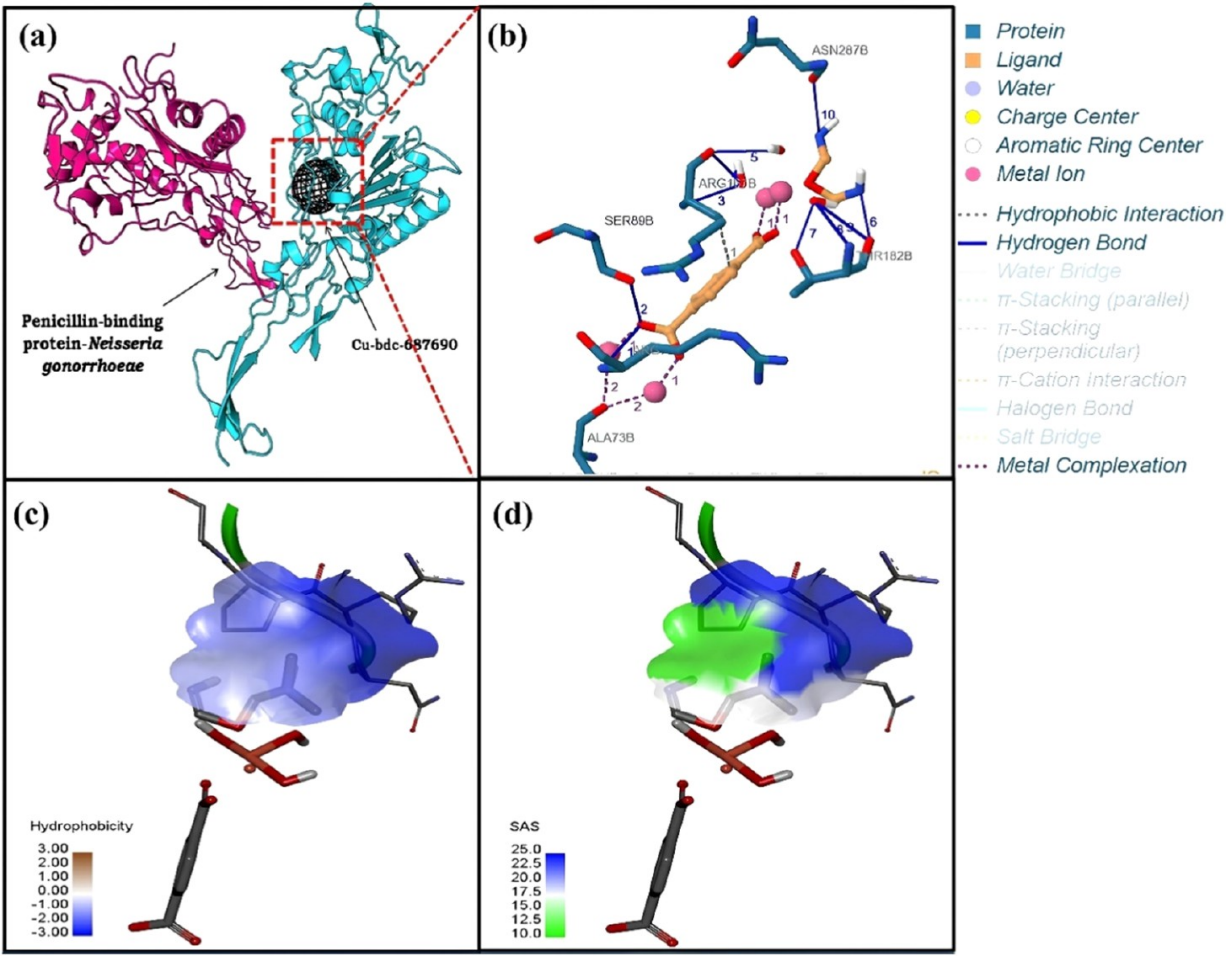
Binding interaction of Cu-BDC and the Ng-PBP2 protein. (a) MD of Cu-BDC with Ng-PB2 representing a ribbon conformation. (b) Crucial molecular interactions at the binding site. (c) Hydrophobicity in the protein–MOF docked complex. (d) Solvent accessibility surface diagram. Two different domains of bacterial protein are represented in red and blue, whereas the MOF is represented by the mesh (black).

**Figure 5:**
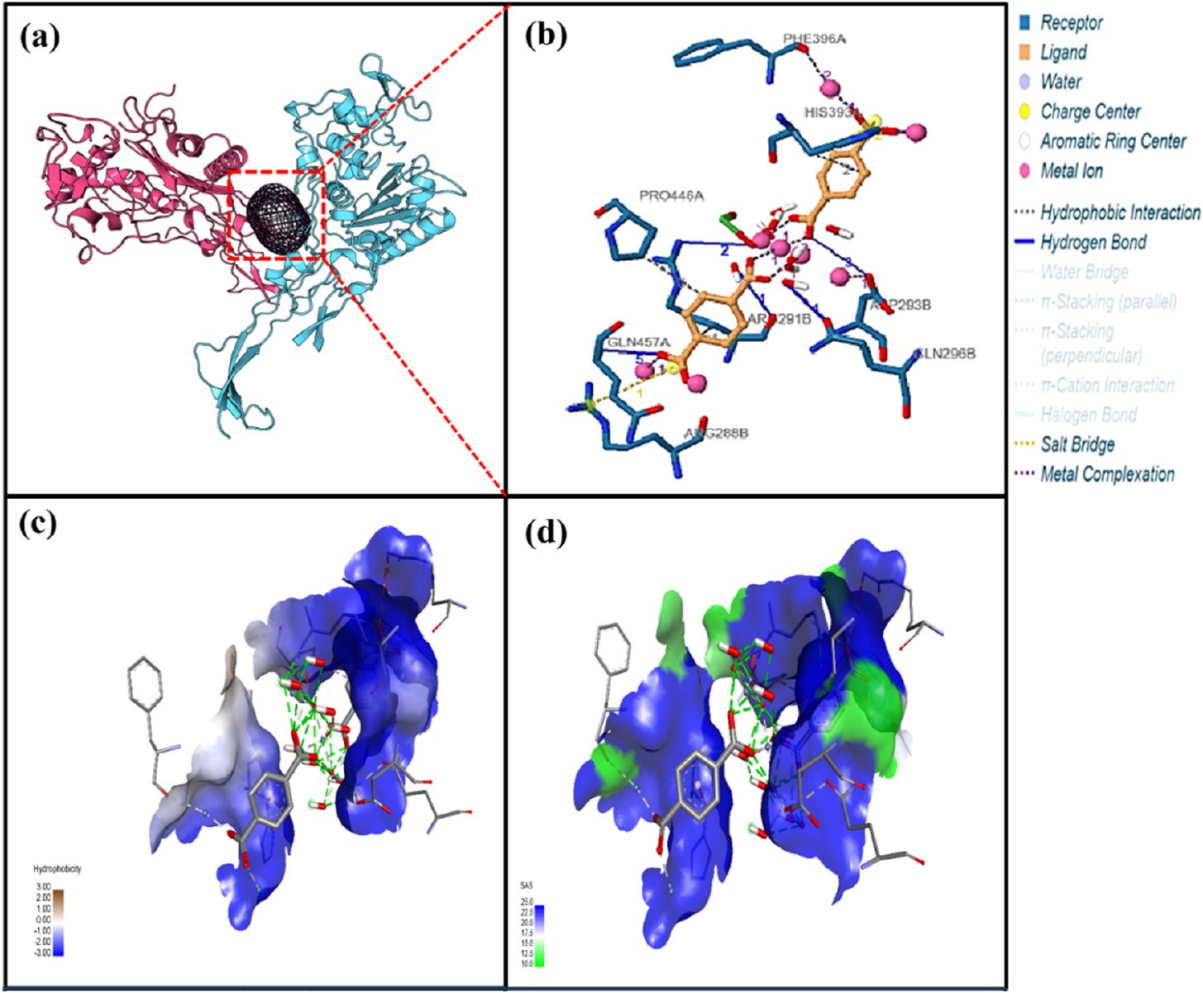
Binding interaction of Ni-BDC and the Ng-PBP2 protein. (a) MD of Ni-BDC with Ng-PBP2 represented with ribbon conformation. (b) Crucial molecular interactions at the binding site. (c) Hydrophobicity in the protein–MOF docked complex. (d) Solvent accessibility surface diagram. Two different domains of bacterial protein are represented in red and blue color, whereas the MOF is represented by the mesh (black).

The images generated using the PLIP web server and depicted in Figures 3–5 visualize the intricate interactions between the MOFs and Ng-PBP2. These visualizations showcased the binding modes of each MOF within the active site crevice of the protein, highlighting key molecular interactions that are crucial for their binding affinity. Furthermore, a detailed depiction of the interactions between the metal complexes and Ng-PBP2, along with the involved residues and their respective distances, is summarized in Table S2. Each MOF exhibited a unique pattern of interactions with the target protein, suggesting distinct binding modes and affinities. The analysis of Fe-BDC docking with Ng-PBP2 revealed multiple hydrophobic interactions, hydrogen bonds, and salt bridges, indicating a diverse range of interactions contributing to the stability of the complex. Similarly, Cu-BDC and Ni-BDC demonstrated significant interactions with Ng-PBP2, including hydrophobic contacts, hydrogen bonds, and metal–protein interactions (Figures 3–5 and Table S2).

**Figure 6:**
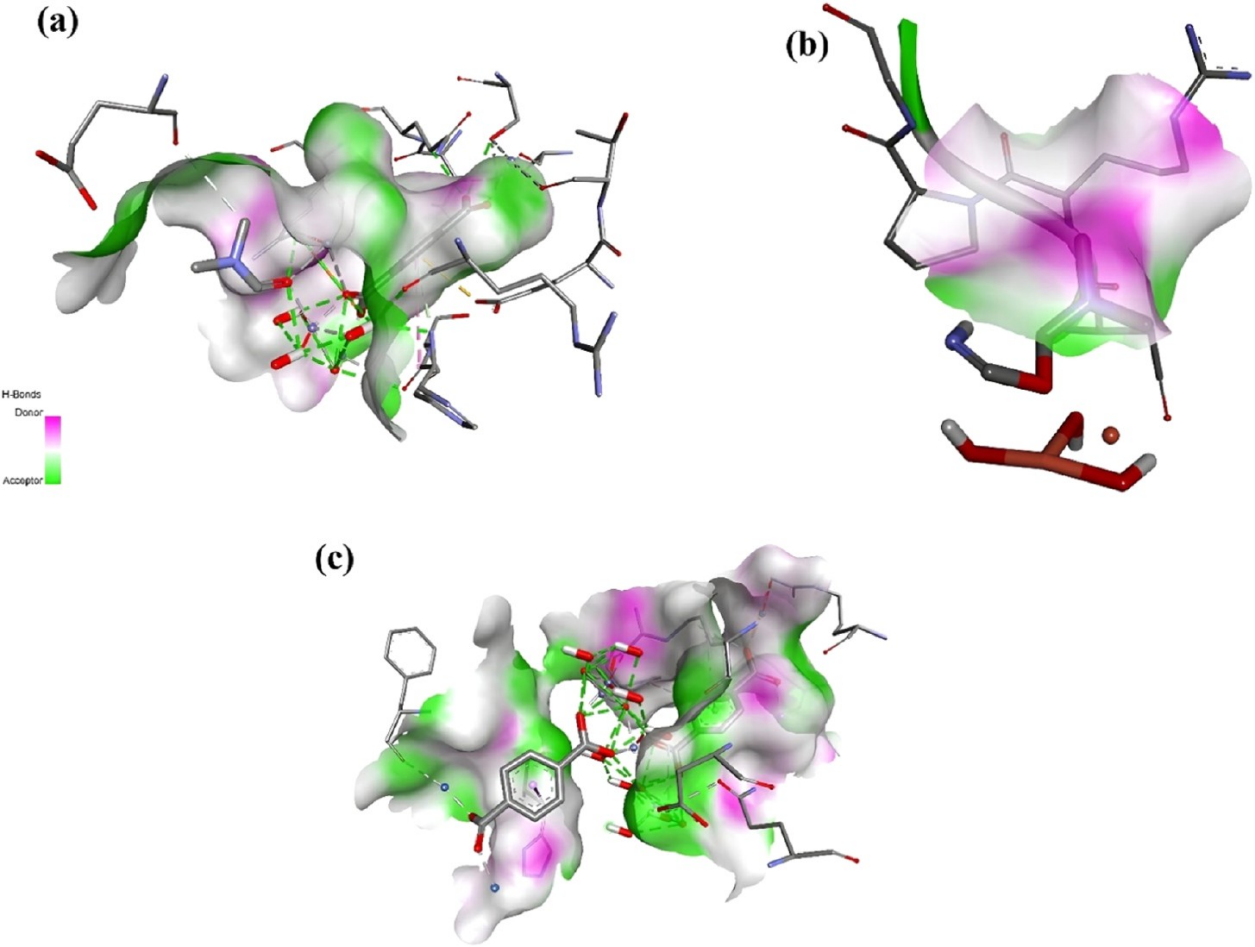
Hydrogen donor–acceptor distribution in Ng-PBP2 docked with different MOFs. This figure depicts the hydrogen-bonding interactions between Ng-PBP2 and MOFs: (a) Fe-BDC, (b) Cu-BDC, and (c) Ni-BDC. In the visualizations, donor sites are indicated in pink, and acceptor sites are in green, with lines representing the hydrogen bonds formed. Both Fe-BDC (a) and Ni-BDC (c) exhibit a significant network of hydrogen bonds, reflecting a strong interaction potential. In contrast, Cu-BDC (b) forms comparatively fewer hydrogen bonds with Ng-PBP2 despite demonstrating the highest binding affinity. This might be due to the binding of Cu-BDC with Ng-PBP2 driven by other interactions, such as π–π stacking or metal coordination, which are not captured in the hydrogen bond analysis. The differences in the hydrogen-bonding patterns among the three MOFs are consistent with their distinct binding mechanisms and affinities.

**Figure 7:**
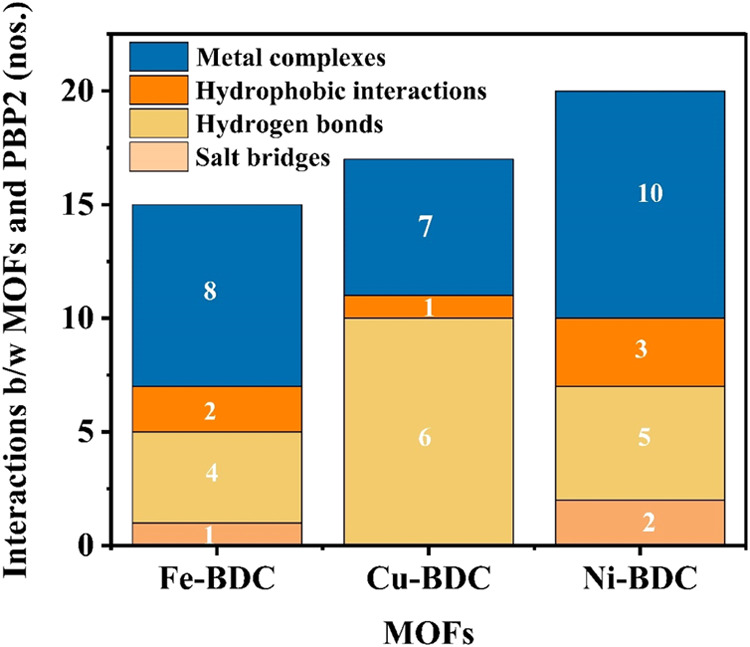
Array showing different interactions and bonding patterns between Fe-BDC, Cu-BDC, and Ni-BDC MOFs and the Ng-PBP2 protein.

Additionally, the hydrophobicity analysis and solvent accessibility surface diagrams provided further insights into the interaction dynamics, highlighting regions of significant chemical interactions and potential binding sites. The hydrogen donor–acceptor distribution analysis illustrated the distribution of hydrogen-bonding interactions, with Cu-BDC displaying a notably higher number of hydrogen bonds, indicative of a strong binding affinity (Figure 6). Moreover, the formation of metal–protein complexes between coordination metal ions and functional groups of amino acids within the proteins further reinforced the stability and specificity of the MOF–protein interactions. The array of different interactions and bonding patterns between Fe-BDC, Cu-BDC, and Ni-BDC MOFs with the target Ng-PBP2 protein is shown in Figure 7. Hydrophobicity is one of the inhibiting factors during binding interactions, and the hydrophobic interactions hinder the functioning of the enzyme system, blocking them from performing relatively specific enzymatic activities. As shown in Figure 5c, Cu-BDC showed the lowest number of hydrophobic interactions in the regions (depicted by the brown color), followed by Fe-BDC (Figure 3c) and Ni-BDC (Figure 4c). Also, solvent accessibility surface area (SASA) is an important parameter for the interaction of accessible surface area available to ligand binding.^50^ The SASA diagrams corresponding to Fe-BDC, Cu-BDC, and Ni-BDC are depicted in Figures 3d, 4d, and 5d, respectively. Ni-BDC MOF showed the maximum SASA area, offering a higher number of binding sites. All of the ligands under investigation displayed significant SASA regions (shown in blue), signifying considerable chemical interactions. These interactions have always been an effective approach in determining the best inhibition process (Figures 6 and 7).^51^

Fe-BDC interactions occurred mainly with chain B, even though chain A is considered the prime chain, and chain B is considered a side chain in the Ng-PBP2 structure. Metal complexes with the Ng-PBP2 protein were formed involving the MOF and active sites of protein atoms in chain B. As shown in Table S2, these complexes had different coordination geometries and bond lengths. Also, there were two hydrophobic interactions with the target protein with residues Leu429A and Gln430A. Many hydrogen bonds were also formed between the MOFs and protein chain B, suggesting a diverse range of interactions.

In the case of interactions between Cu-BDC and Ng-PBP2 (Figure 4 and Table S2), a single hydrophobic interaction was noted with protein residue Arg167B. However, multiple hydrogen bonds were formed involving residues Arg75B, Ser89B, Thr182B, Asn287, and others. This indicated a wide range of interactions. Furthermore, several metal complexes involving Cu atoms interacted with Ala73B, and they exhibited different coordination geometries including trigonal pyramidal and linear arrangements.

In the case of interactions between Ni-BDC and Ng-PBP2 (Figure 5 and Table S2), the MOF engaged in hydrophobic interactions with protein residues Arg291B, His393A, and Pro446A, suggesting multiple binding sites. Also, multiple hydrogen bonds were formed, primarily involving Arg291B, Asp293B, Gln296B, and other residues. Salt bridges were established with Arg88B and His393A in the MOF, interacting with specific carboxylate groups in the ligand. Several Ni-containing complexes were observed in our study, exhibiting both linear and nonlinear coordination geometries such as Asp293B and Phe396A. As per reports, Phe396A was one of the crucial residues that strengthen the Ng-PBP2 protein–ligand interaction.^52^

### Synthesis and Characterization of MOFs

**Figure 8:**
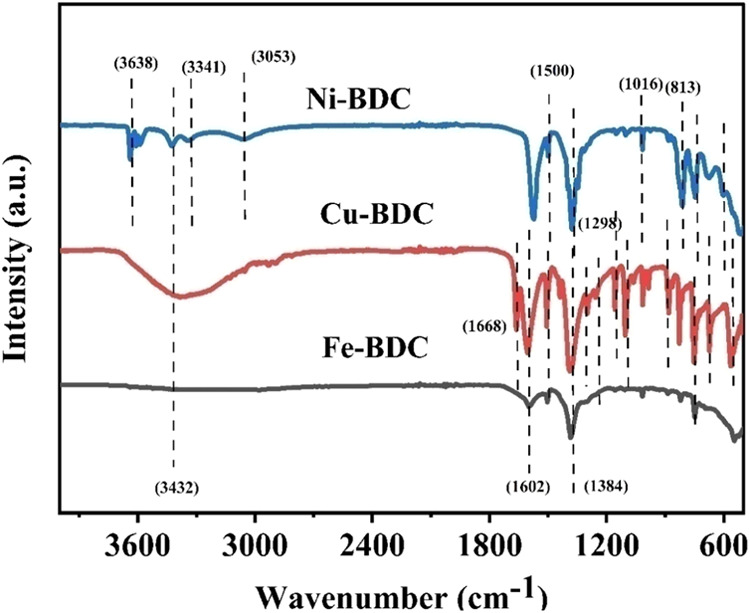
FTIR spectra of Fe-BDC, Cu-BDC, and Ni-BDC MOFs. The figure highlights the characteristic wavenumber ranges for Fe-BDC, Cu-BDC, and Ni-BDC MOFs in FTIR spectra.

FTIR was used to investigate the fingerprints of the molecular vibrations present in the prepared samples. The various functional groups present in Ni-BDC, Cu-BDC, and Fe-BDC MOFs form strong metallic bonds, hydrophobic interactions, and hydrogen bonds with different binding sites of the protein, which might be helpful for the successful inhibition of *N. gonorrhoeae*. In Figure 8, the Fe-BDC MOF curve showed bands at 1312 and 1602 cm^–1^, associated with the carboxylate ligand, validating the coordination of the H_2_-BDC (C_8_H_6_O_5_) linker to the Fe sites.^53,54^ The two pronounced FTIR peaks in the spectra at 1501 and 1384 cm^–1^ were assigned to asymmetric and symmetric vibrations of carboxyl groups (C–O), respectively, validating the existence of the dicarboxylate linker. The bands at 1157 and 748 cm^–1^ were due to C=C and C–H vibration, respectively. In the case of Cu-BDC MOF, an abundant number of functional groups could be observed. The peak at 3432 cm^–1^ was attributed to the C–H stretching vibration arising from the BDC linker. The strong and weak bands at 1500 and 1668 cm^–1^, respectively, were attributed to the remnants of C=O stretching arising from the C–C skeletal vibration of the aromatic ring and carbonyl C=O of BDC. The strong band positioned at 1384 cm^–1^ could be attributed to the stretching vibration of C–O. Additionally, bands in the region 810–1150 cm^–1^ could be attributed to asymmetric and symmetric stretching vibrations of O–C=O, whereas the vibration bands at around 800 cm^–1^ were assigned to Cu–O–Cu, Cu–O, and O–Cu–O. This confirmed that the residues at 1A, 293B, and 396A (Table S2) in protein docked with copper metal complexes might occur *via* strong covalent bonding. In the case of the Ni-BDC MOF curve (Figure 8), the peaks at 3638, 3432, 3341, and 3053 cm^–1^ were assigned to the stretching vibration of OH^–^, COO^–^, and para-aromatic CH^–^ groups, respectively. Two pronounced peaks at 1573 and 1372 cm^–1^ were associated with asymmetric (−COO^–^) and symmetric (−COO^–^) vibrations, respectively.^40,55,56^ The peaks at 1102 and 1091 cm^–1^ were due to C–O stretching, whereas the vibration bands at 816–516 cm^–1^ were associated with Ni–O–Ni, Ni–O, and O–Ni–O. There were a greater number of interactions in the metal–protein complex in Cu-BDC compared to those in Fe- and Ni-BDC, which would require validation by further experimental testing.

**Figure 9:**
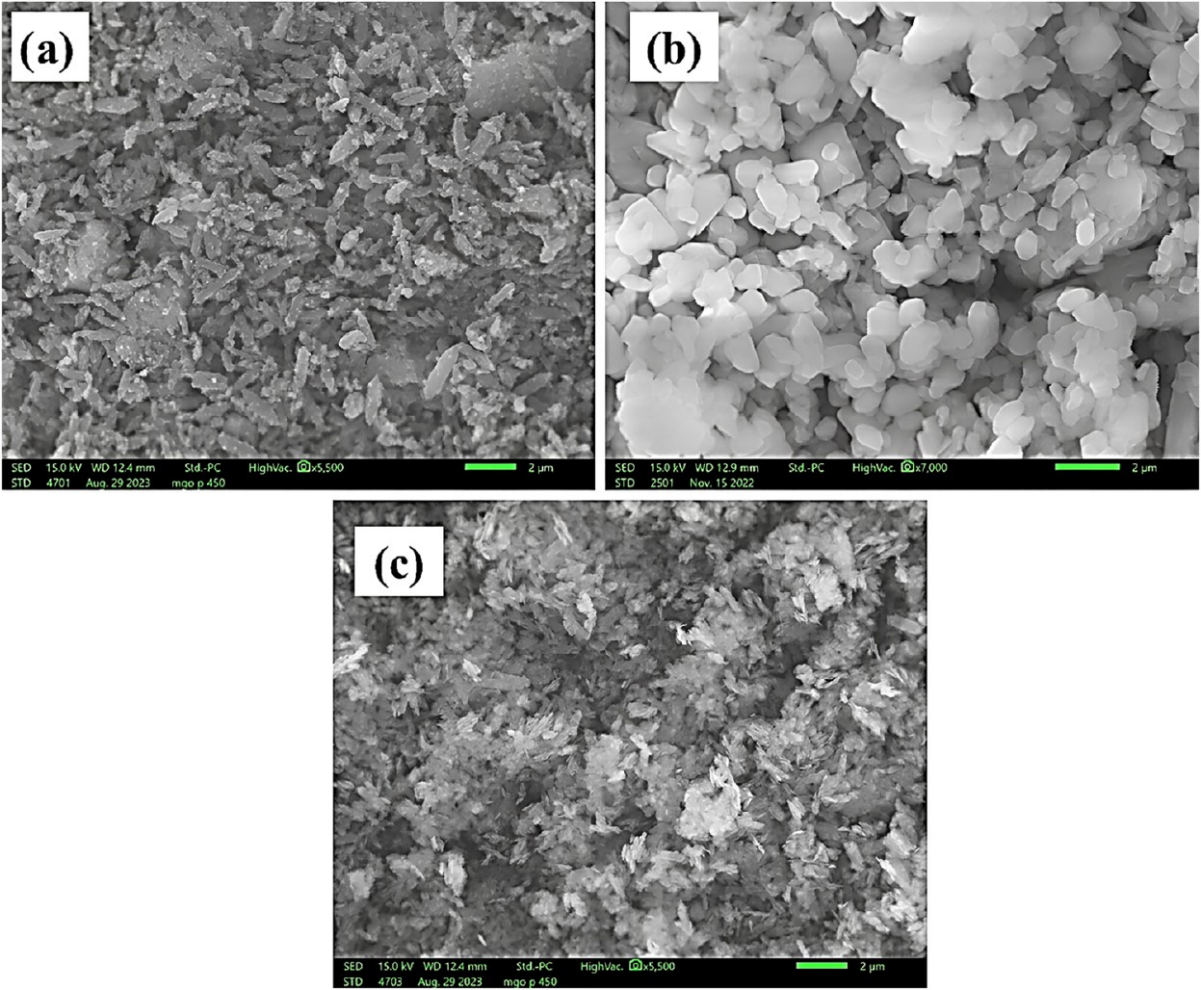
SEM micrographs of Fe-BDC, Cu-BDC, and Ni-BDC MOFs. The figure shows the morphology of the metal–organic frameworks: (a) Fe-BDC with an irregular rodlike structure, (b) Cu-BDC MOF with a large flakelike structure, and (c) Ni-BDC MOF with a dense flakelike appearance.

SEM was used to analyze the morphologies of the prepared MOFs. The Fe-BDC MOF (Figure 9a) demonstrated an irregular rodlike structure with a pointed end; the Cu-BDC MOF showed similar large flakelike structures, providing a larger area for the interaction (Figure 9b), while the Ni-BDC MOF showed a dense flakelike structure (Figure 9c). It was anticipated that the hierarchical arrangement in Ni-BDC, Cu-BDC, and Fe-BDC MOFs substantially increased the porosity and active sites of the material, providing ample space to house other bioactive species, leading to stronger MOF–protein interaction and enhanced antibacterial activity.

**Figure 10:**
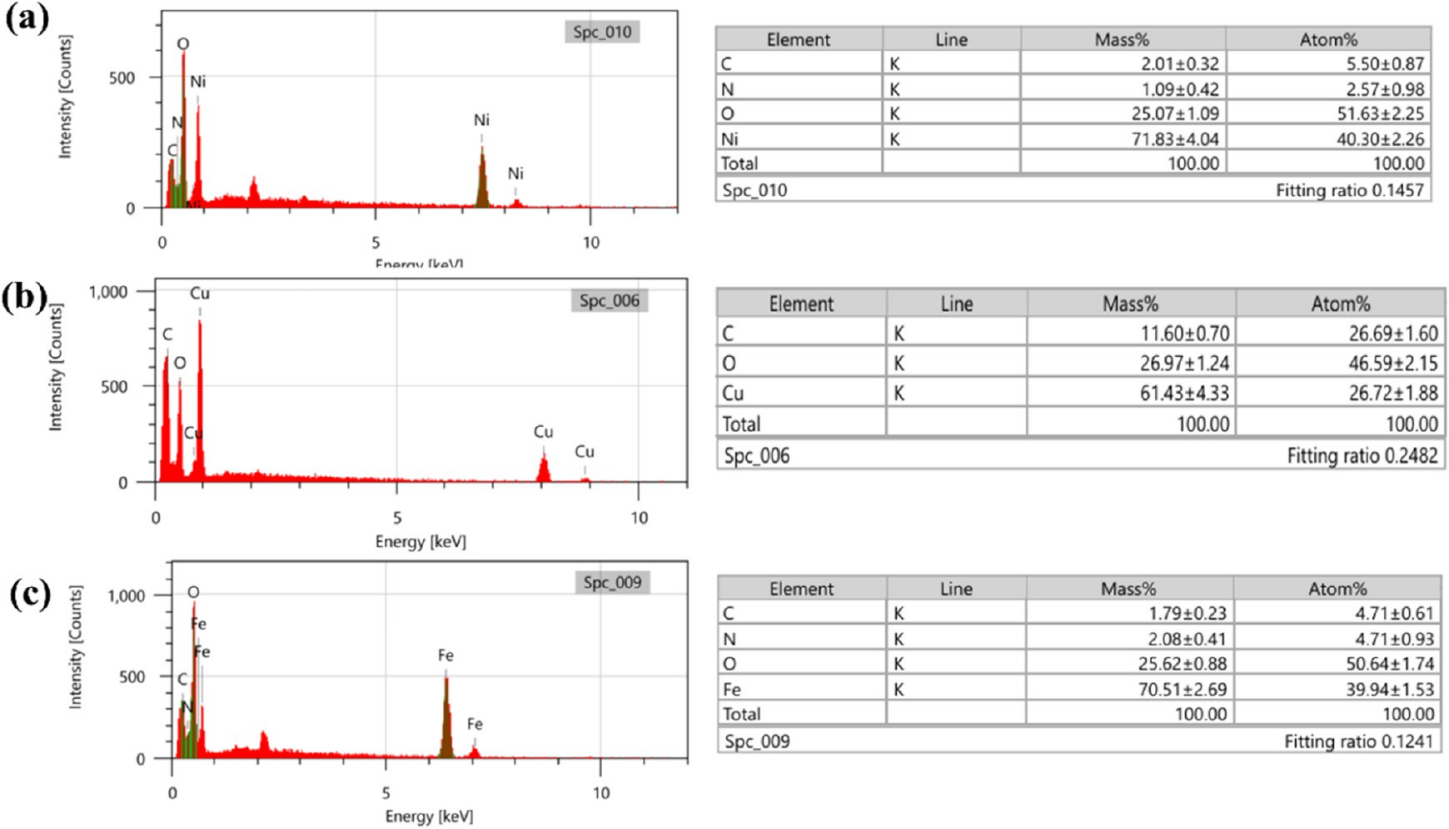
EDS patterns for (a) Ni-BDC, (b) Cu-BDC, and (c) Fe-BDC.

Furthermore, the chemical composition of Ni-BDC, Cu-BDC, and Fe-BDC was examined utilizing energy-dispersive spectroscopy (EDS). The EDS pattern for Ni-BDC (Figure 10a) confirmed the existence of nickel, oxygen, carbon, and nitrogen. The EDS pattern for Cu-BDC (Figure 10b) confirmed the existence of copper, oxygen, and carbon, and for Fe-BDC (Figure 10c), the EDS pattern confirmed the existence of iron, oxygen, carbon, and nitrogen, validating the successful synthesis of materials without any contamination.

**Figure 11:**
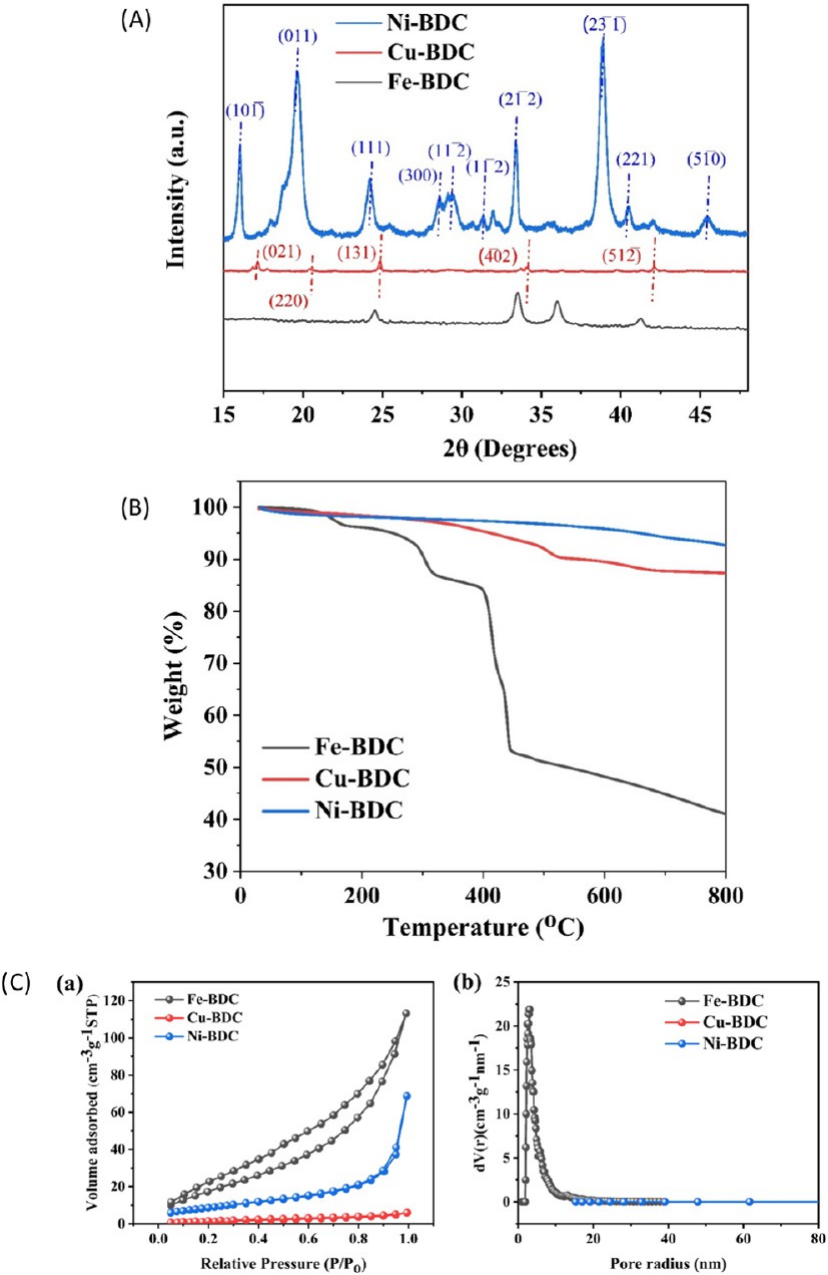
(A) Comparative XRD curves of the Fe-BDC, Cu-BDC, and Ni-BDC MOFs. (B) Comparative thermogravimetric analysis (TGA) patterns of Fe-BDC, Cu-BDC, and Ni-BDC. (C) Comparative (a) N_2_ adsorption and desorption isotherms and (b) pore size distribution curves of Fe-BDC, Cu-BDC, and Ni-BDC MOFs.

X-ray diffraction (XRD) is an efficient technique for examining the crystalline characteristics of materials. The comparative XRD patterns of Fe-BDC, Cu-BDC, and Ni-BDC MOF are shown in Figure 11A, with the samples formed in the synthesis used directly without any alterations. The XRD pattern of Ni-MOF demonstrates its highly crystalline nature compared to Cu-BDC and Fe-BDC. For the Ni-BDC sample, Bragg diffraction peaks were observed at 2θ = 15.9, 19.6, 23.5, 24.2, 28.4, 29.46, 31.3, 33.5, 38.89, 40.6, and 45.01°, attributed to the (101̅), (011), (020), (111), (300), (112̅), (112̅), (21̅2̅), (23̅1̅), (221), and (51̅0) planes of the crystal, respectively. The nickel atom was likely surrounded in an octahedral arrangement by six oxygen atoms, which originated from the BDC ligands or hydroxyl group.^56−59^ For Cu-BDC, Bragg diffraction peaks were observed at 2θ = 17.16, 20.6, 24.84, 34.10, and 42.14° with a few low-intensity peaks. The six diffraction peaks were attributed to the (021), (220), (131), (4̅02), and (512̅) planes of the crystal, respectively. The structure is expended on the crystal plane (021), and Cu^2+^ was connected with BDC^2–^ to form two-dimensional (2D) layered sheets.^41,60,61^ In the case of Fe-BDC MOF, Bragg diffraction peaks were observed at 2θ = 24.4, 33.5, 35.8, and 41.3°, which matches with the reported literature.^62,63^

The thermal stability of the Fe-BDC, Cu-BDC, and Ni-BDC MOFs was assessed through thermogravimetric analysis (TGA) under a nitrogen atmosphere. The analysis was done at varying temperatures at a heating rate of 10 °C/min, as shown in Figure 11B. This technique evaluated the weight percentage changes of the sample as the temperature increased, providing insight into its thermal behavior. For Fe-BDC, weight loss occurred in three distinct stages. The first stage, observed between 60 and 170 °C with a 5% weight reduction, was attributed to the evaporation of trapped water molecules and the breakdown of oxygen-containing functional groups. The second stage, occurring from 170 to 317 °C with a 9.27% weight loss, was due to the structural collapse of Fe-BDC as the ligand decomposed. Finally, the third stage, between 317 and 447 °C with a 34% weight loss, resulted from the progressive decomposition of the framework accompanied by the reduction of iron.^64^ The TGA thermograms of Cu-BDC and Ni-BDC revealed that approximately 87 and 92% of their weight, respectively, remained stable up to 800 °C. This indicated that the synthesized materials exhibited excellent thermal stability, making them suitable for high-temperature applications.

The Brunauer–Emmett–Teller (BET) method was used to examine the specific surface area and porous structure of synthesized samples Fe-BDC, Cu-BDC, and Ni-BDC.^42^ The shape of pores and the type of hysteresis loop are closely related. In mesoporous materials, capillary condensation leads to the appearance of a hysteresis loop, which reflects the disparity between the adsorption and desorption processes.^26,42^ A typical IV-type curve with a distinctive hysteresis loop in the range of 0.04–0.99 *P*/*P*_0_ was obtained, as shown in Figure 11C(a). The specific surface areas of Fe-BDC, Cu-BDC, and Ni-BDC samples were found to be 72, 5.9, and 19.09 m^2^/g, respectively. The pore size distribution is depicted in Figure 11C(b) and highlights the existence of both micropores and mesopores within the structure.

### Antimicrobial Testing of MOFs

**Figure 12:**
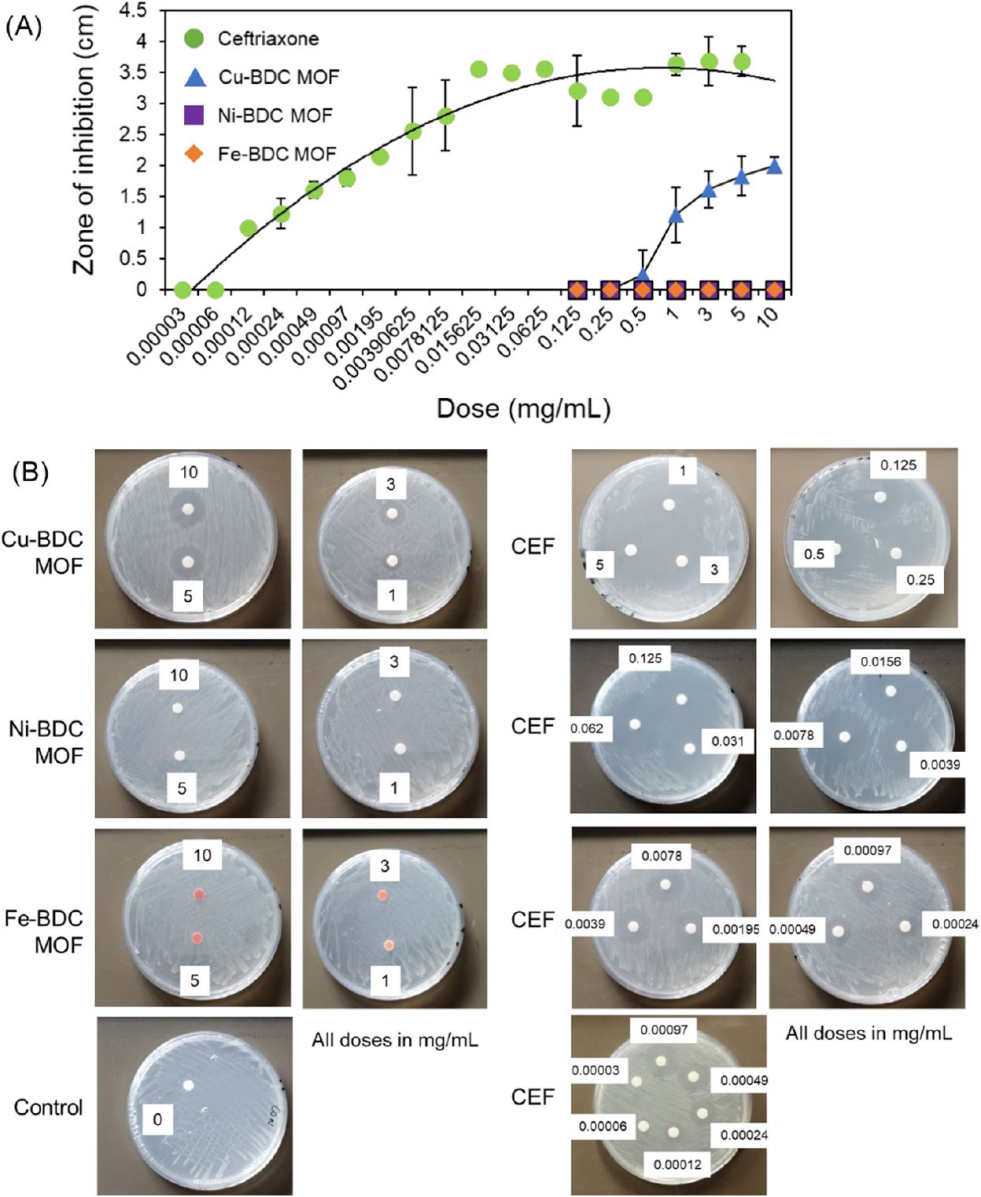
(A) Titration of MOFs and ceftriaxone against *N. gonorrhoeae* strain P9-17. Doses of MOF compounds and the antibiotic ceftriaxone were tested in agar diffusion disk assays against strain P9-17 and the zones of inhibition (ZOIs, diameter in centimeter) were measured after overnight incubation. Symbols represent the ZOIs, and the error bars the standard deviations from at least *n* = 3 experiments. (B) Agar disc diffusion assays for MOFs and ceftriaxone against *N. gonorrhoeae* strain P9-17. Images are representative of experiments done at least *n* = 3 for each compound. Zones of inhibition (ZOIs) are clear around the discs, compared to the control (no compound or diluent alone).

**Figure 13:**
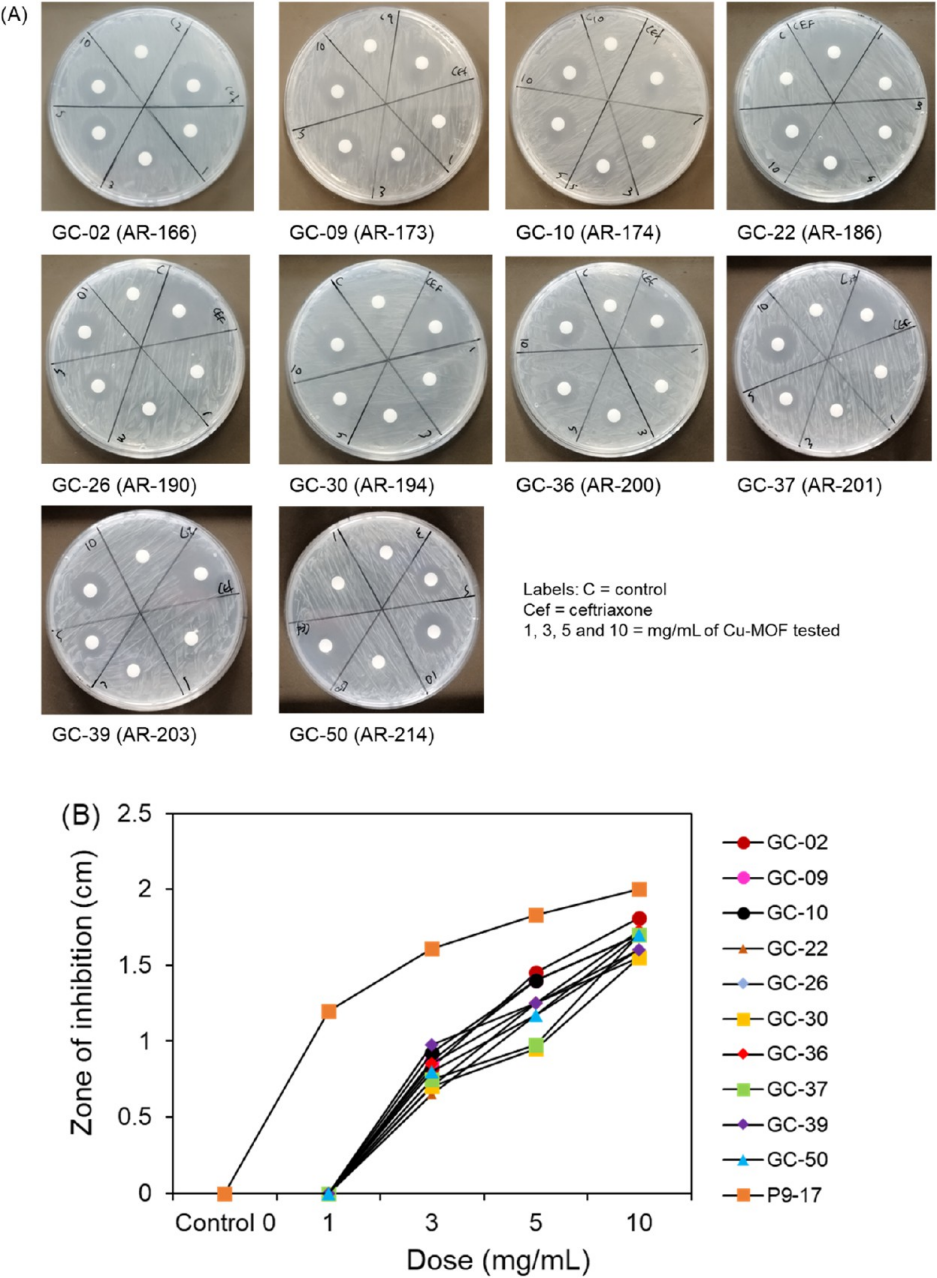
(A) Agar disc diffusion assays for the Cu-BDC MOF tested against gonococcal isolates from the CDC/FDA AR bank of resistant bacteria. The Cu-BDC MOF was tested against those isolates in the bank with the highest MIC values for ceftriaxone and zones of inhibition measured after overnight incubation. Images are representative of *n* = 2 experiments. (B) Titration of the Cu-BDC MOF against gonococcal isolates from the CDC/FDA AR bank of resistant bacteria. Zone of inhibition data for each isolate from panel (A) were plotted against the dose of Cu-BDC MOF tested, compared with sensitive strain P9-17.

The microbicidal activity was assessed by using a standard EUCAST agar diffusion assay. Initially, the antibiotic ceftriaxone (the frontline treatment for uncomplicated gonorrhea^65,66^), and the MOF compounds were titrated against strain *N. gonorrhoeae* strain P9-17, and the zone of inhibition (ZOI) diameters were measured. Of the three metal compounds, both Ni-BDC and Fe-BDC MOF were inactive, with no ZOIs visible even at doses of 10 mg/mL tested (Figure 12A,B). By contrast, the Cu-BDC MOF did show activity, with ZOIs visible at 1, 3, 5, and 10 mg/mL doses (Figure 12A,B). By contrast, ceftriaxone was highly active against P9-17, with ZOIs recorded down to a dose of 0.12 μg/mL (Figure 12A,B). Next, we tested the ability of the active MOF, Cu-BDC MOF (1–10 mg/mL doses), to inhibit the growth of gonococci belonging to the CDC/FDA AR bank, testing the compound against those isolates with the highest reported MIC for ceftriaxone (0.125 μg/mL, CDC/FDA). The Cu-BDC MOF was able to kill all the isolates at doses of 3, 5, and 10 mg/mL (Figure 13A); however, isolate P9-17 was more sensitive with larger ZOIs, and killing was recorded at 1 mg/mL (Figure 13B).

**Figure 14:**
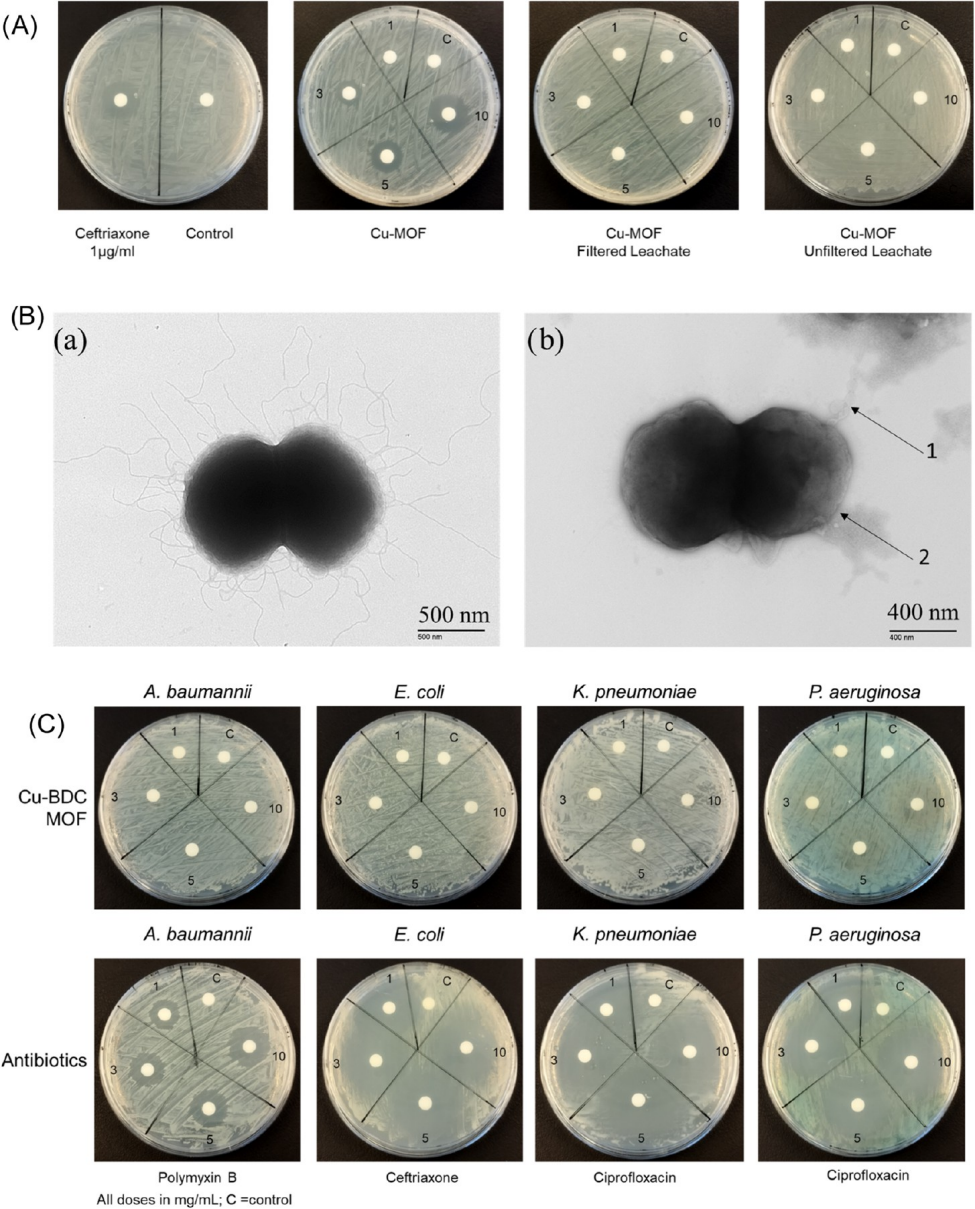
(A) Activity of Cu-MOF leachates against *N. gonorrhoeae* P9-17. Leachates of the Cu-MOF were prepared by centrifugation and filtering of particles suspended in water. Positive controls were Cu-MOF-tested at 1–10 mg/mL and ceftriaxone-tested at 1 μg/mL, and negative controls were water alone. Unfiltered leachate was also tested. The experiments were repeated three times, and representative images are shown. (B) Representative TEM images of (a) untreated *N. gonorrhoeae* P9-17 and (b) *N. gonorrhoeae* treated with the Cu-BDC MOF. Arrow 1 denotes outer membrane shedding, and arrow 2 denotes leaching of cytosol through the membrane. (C) Agar disc diffusion assays for the Cu-BDC MOF and control antibiotics tested against other Gram-negative bacteria. Images are representative of *n* = 3 experiments. Bacteria were grown on NA plates, and discs containing 1–10 mg/mL Cu-BDC MOF or antibiotics were added to their surfaces. Zones of inhibition were examined after an overnight culture.

It is possible that the bactericidal activity of the Cu-MOF for gonococci is due to the leaching of Cu ions from the MOF. To test this hypothesis, we produced unfiltered and filtered leachates from the Cu-MOF suspended in water and tested them for biological activity against gonococci using the standard agar disk diffusion assay. As shown in Figure 14A, neither the unfiltered or filtered leachates showed bactericidal activity compared to the Cu-MOF and ceftriaxone. We also examined the effects of Cu-BDC MOF treatment of gonococci with TEM (Figures 14B and S1). The control shows the classical diplococcal shape of the gonococcus with pili extending from the surface. After treatment, there was a loss of piliation and evidence of cell membrane damage and the leaking of the outer membrane (OM) material and cytosol. To examine the specificity of the reactivity of the Cu-BDC MOF for gonococci, we next tested the compounds against several other Gram-negative bacteria that are important causes of human infections. We tested the Cu-BDC MOF against the ESKAPEE pathogens *A. baumannii*, *E. coli*, *K. pneumoniae*, and *P. aeruginosa*, all of which are in the WHO Priority Pathogen list for the development of new antimicrobials. As shown in Figure 14C, the Cu-BDC MOF showed no bactericidal activity against any of these pathogens in agar disk diffusion assays, even at 10 mg/mL concentration, whereas the appropriate antibiotics demonstrated significant zones of inhibition.

**Figure 15:**
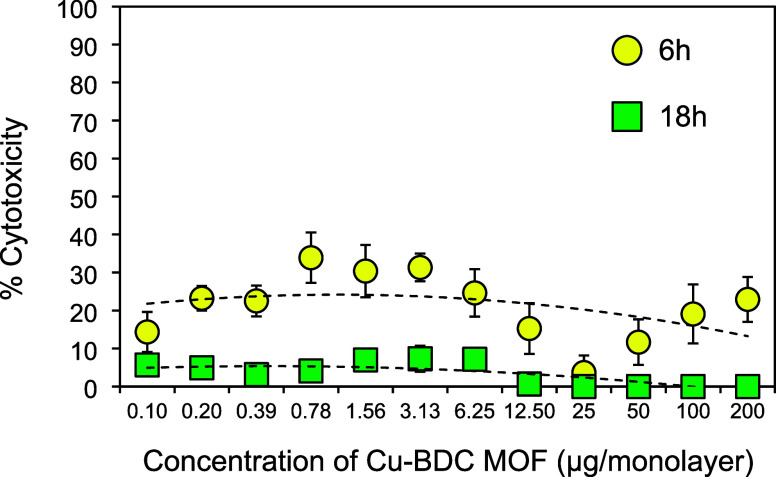
Cytotoxicity of the Cu-BDC MOF for human Chang conjunctival epithelial cells. The symbols represent the means, and the error bars denote the standard errors of the means for *n* = 6 independent experiments with 4 different batches of the Cu-BDC MOF.

Finally, to examine the cytotoxicity of Cu-BDC MOF, human Chang conjunctival epithelial cells, which have been used extensively in gonococcal research, were treated with a range of doses of the Cu-BDC MOF for 18 h. Cytotoxicity was measured at 6 and 16 h after the addition of resazurin, and the percentage of cytotoxicity across the doses averaged ∼20% at the former time point and ∼5% at the latter time point (Figure 15).

## Discussion

The innovativeness of our study is the production of different metal–organic framework compounds (Fe-BDC, Cu-BDC, and Ni-BDC) and their testing, for the first time, *in vitro* against sexually transmitted pathogen *N. gonorrhoeae*. The study shows further innovation in hypothesizing that a gonococcal molecule is putatively targeted by these MOFs. We hypothesized that the target molecule could be penicillin-binding protein 2 (PBP2), located in the gonococcal periplasm, and we then used computational methods to examine the MOF–target protein interactions *in silico*. The rationale for suggesting Ng-PBP2 is that (i) it is a target for β-lactam antibiotics, thus raising the question as to whether it could be targeted by other molecules, and (ii) the crystal structure has been solved,^8^ thus enabling molecular docking (MD) studies. Accordingly, our *in silico* MD analyses indicated a complex interplay of interactions, with the MOFs exhibiting strong metallic bonding, hydrogen bonding, hydrophobic interactions, and covalent bonding with Ng-PBP2. These interactions underscored the ability of Fe-BDC, Ni-BDC, and Cu-BDC MOFs to disrupt the *in silico* binding of Ng-PBP2. The FTIR analysis provided detailed insights into the functional groups and molecular vibrations of the synthesized MOFs, which play a critical role in their interactions with Ng-PBP2. The presence of strong metallic bonds and functional groups such as C=O, C–O, and OH– contributed to the stability and binding efficiency of the MOFs with Ng-PBP2. Furthermore, SEM highlighted the hierarchical and porous structures of the MOFs, which could enhance their potential for containing bioactive species and facilitating stronger interactions with Ng-PBP2.

Despite the promising computational predictions, experimental validation revealed a discrepancy between the *in silico* and *in vitro* results. Initially, we tested the three MOFs against laboratory gonococcal strain P9-17 in a standard minimum bactericidal concentration (MBC) assay, which involved exposure of the compounds to bacteria for 1 h followed by viable counting;^7,12^ however, only the Cu-BDC MOFs demonstrated some bactericidal activity, although the assay was unreliable and not reproducible. Thus, for our study, we used the standard CLSI agar disk diffusion assay because the compounds were relatively insoluble. With this assay, only the Cu-BDC MOF was active, and its ability to kill gonococci appeared to be consistent with the few studies reporting the efficacy of Cu-based antimicrobials against gonococci, *e.g.*, the reported sensitivity to cupric ions^67^ and biogenic copper oxide nanoparticles (CuO NPs).^12^ In contrast, where Fe-BDC and Ni-BDC MOFs showed no bactericidal activity, even at concentrations as high as 10 mg/mL, this lack of effectiveness may stem from limitations such as poor cellular uptake, inadequate surface contact, or instability in the biological environment. Despite their strong docking affinities with Ng-PBP2, the inactivity of these compounds underscores the importance of laboratory evaluation in assessing the biological activity of potential antibacterial agents. Our findings emphasize the limitations of relying solely on computational models for analyzing antimicrobial interactions. Such *in silico* approaches may not fully capture factors like bacterial membrane structure, the effectiveness of MOF interactions, or the role of efflux pumps in diminishing the impact of metal compounds.^52^ Furthermore, a limitation of our study, beyond its current scope, is direct experimental confirmation of the binding of the Cu-BDC MOF with Ng-PBP2, as predicted *in silico*. However, this is not facile: initially, Ng-PBP2 would need to be expressed as a recombinant, leader-free, soluble protein and purified to native conformation. The difficulty is further compounded by the possibility that Ng-PBP2 could be expressed within an insoluble inclusion body. The concern over conformation is important and would require extensive trial-and-error studies to deliver native state protein for biophysical studies, such as isothermal titration calorimetry (ITC), to determine binding affinity.^68^

The utility of Cu-based MOFs as antimicrobials has been demonstrated against other bacteria. Elmehrath et al.^69^ showed that the Cu-1,3,5-benzenetricarboxylate MOF and Cu-deprotonated gallate ligand MOF were inhibitory toward *E. coli* and *Lactobacillus* spp. and inhibition required high concentrations of either compound, up to 2 mg. In their study, the authors used much larger impregnated discs in their disc diffusion assay to assess antibacterial activity (1 cm diameter compared to the CLSI-approved 6 mm discs used in our study) and also reported using a standard broth MIC and a broth time-kill assay to examine the antibacterial properties of their MOFs, although examination of their plates showed possible deposition of the MOFs in the wells and no measurements of bacterial turbidity were provided.^69^ No observation on the solubility of their Cu-based MOFs was provided. The authors suggested a possible mechanism of action that involved damage to the *E. coli* cell membrane (although no visual evidence was provided) and the release of Cu^2+^ ions.^69^

Other Cu-MOFs have also been reported to show microbicidal activity against *Staphylococcus aureus* and *A. baumannii*(70−72) and antifungal activity against *Candida albicans*, *Aspergillus niger*, *A. oryzae*, and *Fusarium oxysporum*.^73^ Xu et al. used an agar well diffusion assay to measure zones of inhibition of a Cu-MOF synthesized using 3,5-dimethyl-1,2,4-triazole and tetrakis (acetonitrile) copper(I) tetrafluoroborate against *S. aureus*, *A. baumannii*, and *E. coli*.^70^ In their paper, the authors tested a significantly higher concentration of 50 mg of MOF per well in their assay, whereas in our study, we tested a maximum dose of 10 mg/mL, which was effective against gonococci but ineffective against *E. coli* and *A. baumannii*. It is possible that 50 mg of Cu-BDC MOF would be needed to see the killing of *E. coli* and *A. baumannii* in our study, although dependency on such concentrations could be viewed as excessive for the development of alternatives to antibiotics. Xu et al. demonstrated a physical contact between their Cu-MOF and the bacteria *via* an electrostatic interaction but did not provide any further evidence on the mechanisms of antibacterial action other than postulating a release of Cu^2+^ ions on the bacterial surface.^70^ Lin et al. produced a two-dimensional Cu-TCPP MOF and a three-dimensional HKUST-1 MOF and used a plate counting method to examine their cidal effects on *E. coli* and *S. aureus*, as well as testing the Cu-MOFs in a *S. aureus* wound infection mouse model.^71^ Surprisingly, the concentrations of MOFs tested against the bacteria were not provided, and both MOFs had poor activity against the bacteria, with only the combination of the HKUST-1 MOF and hydrogen peroxide able to show any bactericidal effect. The mechanism of action was attributed to the peroxidase activity of HKUST-1 generating hydroxyl radicals that are toxic to bacteria. With a viable count assay, the water-soluble HKUST-1 has also been shown to inhibit the growth rate of yeast *C. albicans* and the spore growth of *A. niger*, *A. oryzae*, and *F. oxysporum*,^73^ with concentrations tested up to 500 ppm. Sierra-Serrano developed an agrochemical 2D-MOF called GR-MOF-7, which was based on herbicide glufosinate and Cu^2+^,^72^ which showed good water stability. Using an MBC assay, the authors showed that GR-MOF-7 possessed bactericidal activity against *E. coli* and *S. aureus*, with MBC values between 1 and 2 ppm. Although the potency of our Cu-BDC MOFs cannot be compared directly with HKUST-1 and GR-MOF-7, due to the different bacteria tested and the different assays used, we calculated that 1000–10,000 ppm (1–10 mg/mL) of Cu-BDC was required to kill gonococci. Future side-by-side comparison of the activity of different Cu-MOFs against gonococci could be a selective tool for identifying the best-performing compounds.

The antimicrobial effect of Cu and Cu nanoparticles is well established and is believed to be essentially attributable to the release of Cu^2+^ ions. In our study, a high concentration of the Cu-BDC MOF was required to kill gonococci in the agar disc diffusion assays, which may be a consequence of reduced cellular uptake of the MOF compared, for example, to smaller CuO NPs that can cross bacterial membranes through either ion channels and transporter proteins or diffusion across the membrane directly.^12^ It is possible that the effects of the Cu-BDC MOF are due to uptake by endocytosis or the mechanism of action could be due to the surface contact of Cu-BDC MOF particles with gonococci through a “chelation effect”″ inducing reactive oxygen species, without a significant release of Cu^2+^ ions.^53^ We did observe that treatment with the Cu-BDC MOF resulted in a loss of piliation on the bacterial surface, along with signs of cell membrane damage and the release of the outer membrane and cytosol. A compromised membrane would also enable penetration of the Cu-BDC MOF into the periplasm to interact with Ng-PBP2. These possibilities need to be further explored. Like the hypothesis of Xu et al.,^70^ it is also possible that the interaction of the Cu-BDC MOF with the gonococcal surface and a leaching of Cu^2+^ ions mediate the bactericidal effect. To test this hypothesis biologically, we produced both filtered and unfiltered leachates of the Cu-BDC MOF and showed that neither could kill gonococci in the standard agar disc diffusion assay. We cannot exclude the possibility that the mechanism of gonococcal killing involves Cu^2+^ leaching from the Cu-BDC MOF, although it is possible that only low levels of Cu ions are released from these compounds that do not reach biological efficacy. Cu-BDC MOFs including Cu-BDC-687690 (catena-((μ4-terephthalato)-(*N*,*N*-dimethylformamide)-copper) have been reported to have high stability in aqueous media at different pH levels, which was attributable to the strong coordination interaction between Cu cations and the −COOH group of BDC.^74^ Indeed, EDS analysis of the Cu-BDC MOF prepared in our study validated the presence of copper, oxygen, and carbon, while the XRD pattern highlighted its crystalline nature with distinct peaks corresponding to specific planes, indicating a 2D layered sheet structure. TGA analysis demonstrated high thermal stability, retaining 87% weight up to 800 °C, making it suitable for high-temperature applications. Further, BET analysis revealed a specific surface area of 5.9 m^2^/g with a porous structure containing both micro- and mesopores. These attributes make Cu-BDC MOF a promising material for applications requiring thermal stability and specific surface properties. Thus, it is possible that a consequence of the stability of the Cu-BDC MOF is a slow release of Cu^2+^ ions, and accordingly, future studies could engineer these MOFs to release Cu^2+^ ions more efficiently.

Another significant finding from our study was that the Cu-BDC MOF did not kill a selection of other Gram-negative bacterial pathogens (*A. baumannii*, *E. coli*, and *P. aeruginosa*), demonstrating specificity toward gonococci. Many studies reported that Cu, CuO, biogenically synthesized Cu, nanostructured Cu particles, and Cu coatings exhibit biocidal activity against the other Gram-negative bacteria used in the current study. For example, CuO NPs and Cu-nanowires (CuO-NWs) have been shown to kill *A. baumannii*,^75^ Cu-NPs to kill *E. coli*,^76^ Cu biohybrids with lipase B and sodium phosphate to kill *K. pneumoniae*,^77^ and Cu-based antibacterial coatings to kill *P. aeruginosa*.^78^ Leaching of Cu^2+^ may account for its antibacterial activity, particularly with Cu-based antibacterial coatings;^78^ although we postulate that if this were occurring significantly from the Cu-BDC MOF, then we may have seen some activity against these other Gram-negative pathogens that are susceptible to the effects of Cu^2+^. The pathogen specificity of Cu-BDC MOF could be due to (i) the explicit binding of Cu-BDC with Ng-PBP2, which may disrupt normal cellular homeostasis, or perhaps (ii) differences in the outer membrane (OM) composition of the different bacteria. Although the essential Gram-negative OM of these bacteria is a typical lipid bilayer with large amounts of lipopolysaccharide (LPS) and proteins, the presence of different proteins and LPS structures may play a role in providing resistance of *A. baumannii*, *E. coli*, and *P. aeruginosa* against the Cu-BDC MOF.

We also found low levels of cytotoxicity of the Cu-BDC MOF using a standard cell culture resazurin assay (∼5–20%). This is reassuring, given the concerns regarding the potential accumulation of toxic levels of copper due to leaching.^79^ Our data are consistent with other studies examining the toxicity of copper MOFs, including HKUST-1. This Cu-based MOF was cytotoxic to HEK293 hepatocytes *in vitro* only at high doses of 50–100 μg/mL, and the authors concluded that the toxic risk of this compound is negligible.^80^

## Conclusions

The *in silico* and *in vitro* investigations into the interactions of Fe-BDC, Ni-BDC, and especially Cu-BDC MOFs with the Ng-PBP2 protein highlight the promise of MOFs as candidates for addressing gonococcal infections. Additionally, the unique structural properties of these MOFs suggest their potential for broader applications, including targeted drug delivery and controlled release, particularly for infections affecting exposed mucosae and skin. For example, chitosan membranes loaded with Cu-MOFs and used as dressings were reported to show antibiofilm and proangiogenic properties in a *P. aeruginosa*-infected wound rat model.^81^ In addition, Cu-MOFs have been successfully loaded with various antibiotics such as rifampicin,^82^ nitric oxide,^83^ and chlorhexidine,^84^ leveraging their structure to enable gradual release of these agents. In the case of gonococci, the next stage of development could include extensive studies with the mouse intravaginal model of gonococcal infection^85^ to examine the efficacy of Cu-BDC MOFs. This model could also be useful to study how to deliver Cu-BDC MOF to patients with gonorrhea. However, prior studies would need to (i) examine toxicity further *in vitro* and *in vivo*, (ii) probably involve chemical modification of the compound to increase solubility to improve their efficacy, and (iii) refine their ability to deliver therapeutic agents in a targeted and controlled manner. It is possible that MOFs could be used to construct intravaginal rings^86^ to treat women with mucosal infection, which would require further developmental work. Given the evidence that bacteria can develop resistance to copper,^87^ an examination of the ability of gonococci to build potential resistance to Cu-BDC MOFs would be necessary, for example, by (i) using the hollow fiber model,^88^ (ii) examining the potential role of the gonococcal efflux pumps, and (iii) examining transcriptional changes in the organism’s copper homeostasis mechanisms. The possibility of developing multifunctional MOFs capable of targeting multiple bacterial proteins or pathways can also be explored.

## Data Availability

All data generated in this study are presented in the manuscript and Supporting Information.

## Supporting Information Available

Illustrations of various MOFs, their three-dimensional structures, and reported physicochemical and biological properties (Table S1); detailed depiction of the various interactions between the metal complex and Ng-PBP2, the different residues involved in the interaction, and their respective distances analyzing the crucial interactions involved (Table S2); and additional representative TEM images of *N. gonorrhoeae* P9-17 bacteria (Figure S1) (PDF)
