# Supplementary material for: Computational and Experimental Study of Metal–Organic Frameworks (MOFs) as Antimicrobial Agents against Neisseria gonorrhoeae
Source: ACS Appl Mater Interfaces. 2025 Mar 27;17(14):20628–46. doi: 10.1021/acsami.4c15851 (PMC11986912; doi:10.1021/acsami.4c15851)
Supplement: Supplementary file 1 — am4c15851_si_001.pdf [file am4c15851_si_001.pdf]

Supporting Information

A Computational and Experimental Study of Metal-Organic Frameworks (MOFs) as Antimicrobial Agents Against *Neisseria gonorrhoeae*

Ravi Kant [1, 2 #], Megha Prajapati [3,4 #], Pradip Das [2,5], Daman Saluja [1], Myron Christodoulides [2 *], Chhaya Ravi Kant [3 *]

1. Medical Biotechnology Laboratory, Dr. B. R. Ambedkar Center for Biomedical Research, University of Delhi, Delhi-110007, India
2. Molecular Microbiology, School of Clinical and Experimental Sciences, Faculty of Medicine, University of Southampton, Southampton SO16 6YD, UK
3. Department of Applied Sciences and Humanities, Indira Gandhi Delhi Technical University for Women, Kashmiri Gate, Delhi-11006, India
4. Electronics Materials Lab, College of Science and Engineering, James Cook University, Townsville, QLD, 4811, Australia
5. School of Physics and Astronomy, University of Southampton, Southampton SO17 1BJ, United Kingdom.

# Equal contribution as first authors

*Authors for Correspondence: chhayaravikant@igdtuw.ac.in (C.R.K.) for MOFs; mc4@soton.ac.uk (M.C.) for biological testing.

Table S1: Illustrations of various MOFs, their three-dimensional structures, and reported physico-chemical and biological properties.

S. No.	MOF	Structure	Physio-chemical properties	Biological Activity
1	Ag-BDC-198096-Catena-((μ6-Benzene-1,4-dicarboxylato)-di-silver)[1]	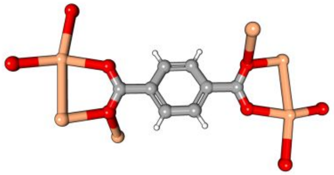	• Biocompatibility and low toxicity<br>• Outstanding luminescence and semiconducting properties.<br>• Affordability compared with rare-earth elements [2]	• Combat 99% of bacterial, growth both in sessile and planktonic state<br>• Antifouling effect<br>• Anti-adherent and biocide properties<br>• Building coatings to achieve bacteria-free surfaces for implants, water treatment, bio corrosion, food packaging, and heat exchange [3]
2	Ag-BTC-631984-Catena-(bis (μ7-Benzene-1,3-dicarboxylato-5-carboxylic acid)-bis (μ6-benzene-1-carboxylato-3,5-dicarboxylic acid)-hexa-silver) [4]	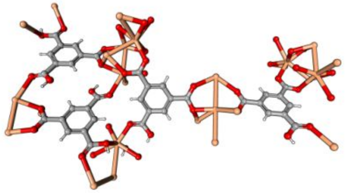	• High Ag dispersion and large electrochemical surface area [5]	• Full inactivation, and degradation of Escherichia coli and Staphylococcus aureus<br>• Biofouling mitigation [6]
3	Bio-MOF-1432054-Catena-(tris(μ-2,2'-((1,2-dihydroxyethane-1,2-diylidene) diazanylidene) dipropionate)-tris(μ-hydroxo)-calcium-hexa-copper dotriacontahydrate) [7]	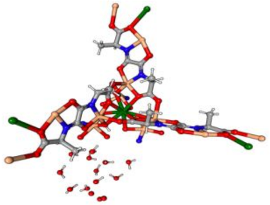	• High Proton Conductivity<br>• Maintain structural stability<br>• Excellent stability in presence of water<br>• Permanent porosity<br>• Chiral structure contributing to stability [8]	• Antimicrobial: Some bioMOFs show antimicrobial properties.<br>• Drug Delivery: Porous structure aids in controlled release of drugs.

				• Biocompatibility: Suitable for biomedical applications, including tissue engineering [9]
4	Co-BDC-153067 Catena-((μ6-Terephthalato)-bis(μ3-hydroxo)-di-cobalt) [10]	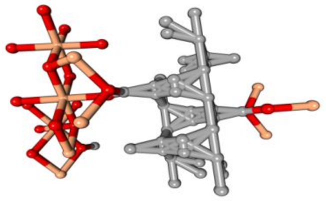	• Readily available [11]	• Inactivation of *Escherichia coli (E. coli)* due to release of metal ions and organic linker<br>• Shows remarkable antioxidant properties that shows autooxidation of adrenaline solution [12]
5	Zn-ZIF-2-602542 Catena-(bis (μ2-2-Methylimidazolato-N, N')-zinc(ii) unknown clathrate hydrate) [13]	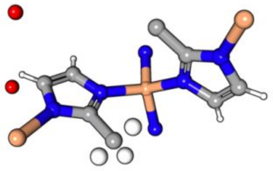	• High thermal stability, large surface area,<br>• High chemical resistance to various solvents [14]	• Remove contamination at low scale.<br>• Removed both oxytetracycline (OTC) and tetracycline (TC) [15]
6	Co-ZIF-671073 Catena- (dodecadic(μ 2-2-methylimidazolato)-hexa-cobalt tetrahydrate clathrate) [16]	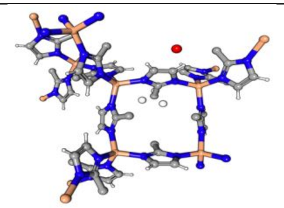	• Highly stable structure validates its high adsorbent's re-usability [17]	• Shows excellent antibacterial properties by control release of Co^{2+} ions which inhibit the growth of bacteria membrane.<br>• Inhibition of *Staphylococcus aureus* and *E. coli* [17]
7	Cr-BDC-605510 Catena- [tris(μ 4-Terephthalato) -(μ3-oxo)-di aqua-fluoro-tri-chromium Penta decahydrate] [18]	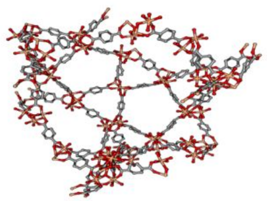	• Utilized in gas storage and separation, phase adsorption, and catalysis.<br>• Have unsaturated Lewis's acid sites in the structure helpful in chemical /thermal/water/chemical stability [19]	• Inhibition of Gram+ve *S. aureus* and Gram -ve *E. coli* [19]

8	Cu-BDC-687690 Catena-((μ4-terephthalato)-(N,N-dimethylformamide)-copper) [20]	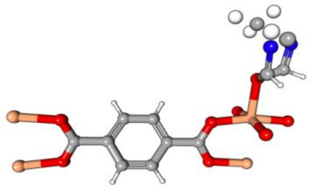	• Non-toxic<br>• Highly stable<br>• Environmentally friendly [21]	• Inhibition of *E. coli* [22]
9	Cu-BTC-112954 Catena-[bis(μ6-Benzene-1,3,5-tricarboxylato)-triaqua-tri-copper decahydrate clathrate] [23]	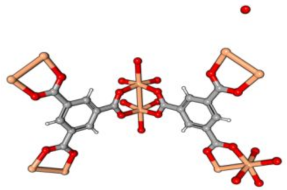	• High redox activity, chemical stability, and photochemical properties<br>• Substantially investigated in wide spectra like hydrogen storage, absorption, HER and CO_2 reduction photovoltaics ammonia sensing [24]	• Antibacterial properties against *E. coli*<br>• Employed in the fabrication of antibacterial clinical fabrics due to its ability of bacteria control [25]
10	Fe-BDC-258445 Catena-((μ4-Benzene-1,4-dicarboxylato) -(μ2-dimethylformamide)-iron) [26]	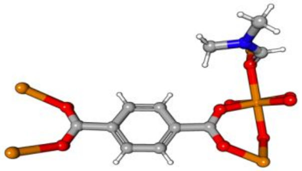	• Working for photocatalyst activity, nontoxic nature, low cost<br>• Utilized in degradation of non-biodegradable organic pollutants<br>• Recyclable [27]	• Inhibition of fungus, bacteria, and yeast.<br>• Antibacterial activity against vancomycin.<br>• Control of the delivery process of, antiviral, antibacterial, antiparasitic, and agents [28]
11	Fe-BTC-640536 catena-(hexakis(μ6-Benzene-1,3,5-tricarboxylato)-tris(μ3-oxo)-hexaaqua-trifluoro-nona-iron hydrate) [29]	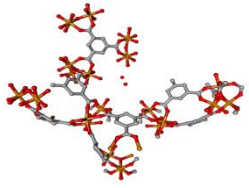	• Have remarkable textural properties generally regarding the external specific surface area [29]	• Excellent antibacterial properties against *E. coli* [30]

12	IRMOF-3-175574 catena-(tris(μ4-2-Aminobenzene-1,4-dicarboxylato) -(μ4-oxo)-tetra-zinc heptakis(N, N-diethylformamide) clathrate) [31]	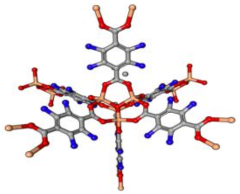	• Possess free amine functionality<br>• Employed in gas-storage applications [32]	• Antibacterial Efficacy: IRMOF-3 has demonstrated effectiveness against various Gram-negative and Gram-positive bacteria.<br>• Gram-Negative Bacteria: Effective against *E. coli* and *Salmonella typhi*<br>• Gram-Positive Bacteria: Effective against *Bacillus cereus* and *Listeria monocytogenes* [32, 33]<br>• Antibiotic Interaction: Functions in combination with antibiotics like amoxicillin, enhancing its antibacterial properties.
13	Ni-BDC-638866 Catena-(bis(μ4-terephthalato)-bis(μ3-hydroxo)-tetra-aqua-tri-nickel dihydrate) [34]	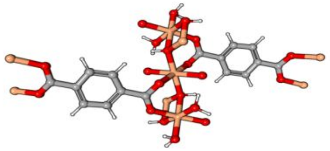	• Very high specific surface area<br>• Tunable pore size<br>• Remarkable thermal/chemical/water stability, which makes it widely used in drug delivery, gas storage and separation, catalysis [34]	• Inhibition of yeast pathogen candida albicans, Gram+ve bacteria *Bacillus subtilis*, Gram-ve bacteria *E. coli* and *Pseudomonas aeruginosa* [35]
14	Ni-BTC-274177 catena-((μ3-Benzene-1,3,5-tricarboxylato)- (μ3-5-carboxybenzene-1,3-dicarboxylato)- (μ3-3,5-dicarboxybenzoato)-hexa-aqua-tri-nickel(ii) dihydrate) [36]	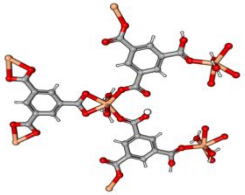	• High conductivity and specific surface area [37]	• Used for quantitative determination of histamine [37]

15	Ni-Hmim-724098 catena-tetrakis(μ2-3-t-Butylimidazolato-2-thiolato)-di-nickel acetonitrile solvate [38, 39]	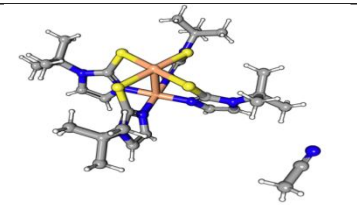	• Structural stability, and electrocatalytic properties [40]	• Larvicidal activity against larvae *A. aegypti* mosquito<br>• Antimicrobial activity against gram-negative bacterial strains [40]
16	Ti-MOF-751157 Catena-(hexakis(μ4-Benzene-1,4-dicarboxylato)-tetrakis(μ2-hydroxo)-octakis(μ2-oxo)-octa-titanium monohydrate) [41]	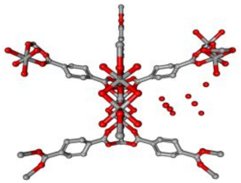	• Involves CO_2 reduction, H_2 and H_2O_2 production, N_2 fixation<br>• Remedial for the removal of inorganic and organic pollutants<br>• Utilized in photocatalytic sensors [42]	• Utilized in the degradation of dye molecules<br>• Destroy the cell walls of bacteria [42]
17	Zn-BDC-256965 catena-(tris (μ4-1,4-Benzenedicarboxylato)-(μ4-oxo)-tetra-zinc octakis(dimethylformamide) chlorobenzene clathrate) [43]	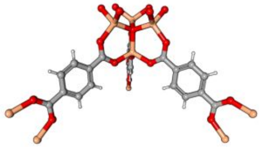	• High yield<br>• Easily synthesized<br>• Non-toxic<br><br>• Economcal [22]	• Inhibition against the Gram+ve strain *S. aureus* and Gram-ve strain *E. coli* [44]
18	Zn-BTC-1962948 Catena-[bis(μ-benzene-1,3,5-tricarboxylato)-triaqua-tri-zinc(ii) unknown solvate hydrate] [38]	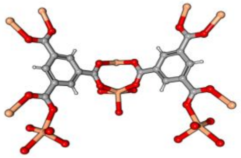<br>90°	• low toxicity<br>• high stability [45]	• Promising capacity to cell migration and proliferation, and lower toxicity to fibroblasts.<br>• Enhance the ability of wound healing genes like collagen with no toxicity to body [45]

19	Zn-ZIF-864311 catena-[dodecakis (µ2-2-Methylimidazolyl)-hexa-zinc(ii) octahydrate] [46]	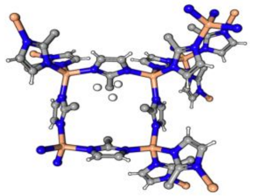	• Utilized in low energy gas separation, sensor technology, and catalytic nano reactor.<br>• Shows high thermal and chemical stabilities good dispersity, and, high loading capacity [14]	• Inhibition of *S. aureus* and *E. coli*<br>• Promotes wound healing<br>• Utilized as nanocarrier for delivery, high loading capacity, chemical drugs, photodynamic agents, RNA-protein CRISPR complexes, nucleic acid therapeutics, proteins [15]

Table S2: Detailed depiction of the various interactions between the metal complex and Ng-PBP2, the different residues involved in the interaction, and their respective distances analyzing the crucial interactions involved.

**Fe-BDC**							
**Hydrophobic Interactions**							
Index	Residue	AA	Distance	Ligand Atom	Protein Atom		
1	429A	Leu	3.93	6390	2247		
2	430A	Gln	3.59	6391	2253		
**Hydrogen Bonds**							
Index	Residue	AA	Distance H-A	Distance D-A	Donor Angle	Donor Atom	Acceptor Atom
1	393A	His	2.83	3.82	165.84	1956[N3]	6370[O3]
2	430A	Gln	3.00	3.36	102.67	2248[Nam]	6397[O3]
3	433A	Arg	3.00	3.89	150.60	2279[Ng+]	6379[N3]
4	433A	Arg	3.10	3.97	147.39	2280[Ng+]	6379[N3]
**Salt Bridges**							
Index	Residue	AA	Distance	Ligand Group	Ligand Atom		
1	433A	Arg	4.25	Carboxylate	6370,6371		
**Metal Complexes**							
Index	Residue	AA	Metal	Target	Distance	Location	
Complex 1: Fe, NA (1)							
3	1A	RES	6372	6370	2.06	Ligand	
Complex 2: Fe, NA (1)							
1	1A	RES	6375	6371	2.06	Ligand	
Complex 3: Fe, Trigonal Pyramidal (3)							
4	1A	RES	6396	6395	2.10	Ligand	
5	400A	Thr	6396	2010	2.65	Protein Main Chain	
6	427A	Ser	6396	2231	2.73	Protein Side Chain	
Complex 4: Fe, Trigonal Pyramidal (3)							

2	1A	RES	6398	6397	2.10	Ligand	
5	301A	Ala	6398	1283	2.32	Protein Main Chain	
7	427A	Ser	6398	2231	2.32	Protein Side Chain	
**Cu-BDC**							
**Hydrophobic Interactions**							
Index	Residue	AA	Distance	Ligand Atom	Protein Atom		
1	167B	Arg	3.01	6378	3417		
**Hydrogen Bonds**							
Index	Residue	AA	Distance H-A	Distance D-A	Donor Angle	Donor Atom	Acceptor Atom
1	75B	Arg	2.78	3.74	163.06	3270[Nam]	6380[O3]
2	89B	Ser	2.47	3.22	133.88	3374[O3]	6380[O2]
3	167B	Arg	2.20	2.95	131.65	3413[Nam]	6397[O3]
4	167B	Arg	2.63	3.04	105.57	6397[O3]	3416[O2]
5	167B	Arg	2.60	3.11	113.36	6399[O3]	3416[O2]
6	182B	Thr	2.18	3.19	168.47	6393[N2]	3532[O2]
7	182B	Thr	2.01	2.60	117.26	3534[O3]	6395[O3]
8	182B	Thr	2.57	3.38	139.52	3529[Nam]	6395[O3]
9	182B	Thr	2.29	2.99	128.65	6395[O3]	3532[O2]
10	287B	Asn	2.76	3.29	112.58	6388[N3]	4309[O2]
**Metal Complexes**							
Index	Residue	AA	Metal	Target	Distance	Location	
Complex 1: Cu, NA (1)							
4	1A	RES	6370	6371	1.94	Ligand	
Complex 2: Cu, linear (2)							
2	1A	RES	6381	6380	1.94	Ligand	
6	73B	Ala	6381	3261	2.54	Protein. Mainchain	
Complex 3: Cu, linear (2)							
1	1A	RES	6383	6382	1.96	Ligand	

5	73B	Ala	6383	3261	2.43	Protein Side Chain	
Complex 4: Cu, Liner (2)							
2	1A	RES	6416	6414	2.12	Ligand	
7	450A	Glu	6416	2409	2.77	Protein. Mainchain	
Complex 4: Cu, NA(1)							
3	1A	RES	6385	6384	1.96	Ligand	
**Ni-BDC**							
**Hydrophobic Interactions**							
Index	Residue	AA	Distance	Ligand Atom	Protein Atom		
1	291B	ARrg	3.80	6392	4340		
2	393A	His	3.86	6402	1960		
3	446A	Pro	3.78	6394	2375		
**Hydrogen Bonds**							
Index	Residue	AA	Distance H-A	Distance D-A	Donor Angle	Donor Atom	Acceptor Atom
1	291B	Arg	2.84	3.73	151.88	6411[O3]	4339[O2]
2	291B	Arg	3.08	3.73	125.06	4345[Ng+]	6381[O3]
3	293B	Asp	2.91	3.76	150.55	4358[O3]	6380[O3]
4	296B	Gln	2.45	3.17	131.42	6387[O3]	4382[O2]
5	457A	Gln	2.27	3.09	139.96	2453[Nam]	6398[O3]
**Salt Bridges**							
Index	Residue	AA	Distance	Ligand Group	Ligand Atom		
1	288B	Arg	5.13	Carboxylate	6396,6398		
2	393A	His	4.25	Carboxylate	6409,64		
**Metal Complexes**							
Index	Residue	AA	Metal	Target	Distance	Location	
Complex 1: Ni, NA (1)							
8	1A	RES	6370	6372	2.05	Ligand	
Complex 2: Ni, Linear (2)							
4	1A	RES	6371	6373	2.03	Ligand	

5	1A	RES	6371	6380	2.03	Ligand	
Complex 3: Ni, NA (1)							
1	1A	RES	6376	6379	2.05	Ligand	
Complex 4: Ni, NA (1)							
9	293B	Asp	6377	4358	2.45	Protein Side Chain	
Complex 5: Ni, NA (1)							
7	1A	RES	6397	6396	2.03	Ligand	
Complex 6: Ni, NA (1)							
2	1A	RES	6399	6398	2.03	Ligand	
Complex 7: Ni, NA (1)							
6	1A	RES	6408	6407	2.03	Ligand	
Complex 8: Ni, Linear (2)							
3	1A	RES	6410	6409	2.03	Ligand	
10	396A	Phe	6410	1979	2.77	Protein Main Chain	

Figure S1. Additional representative TEM images of *N. gonorrhoeae* P9-17 bacteria a) untreated and b-d) treated with Cu-BDC MOF.

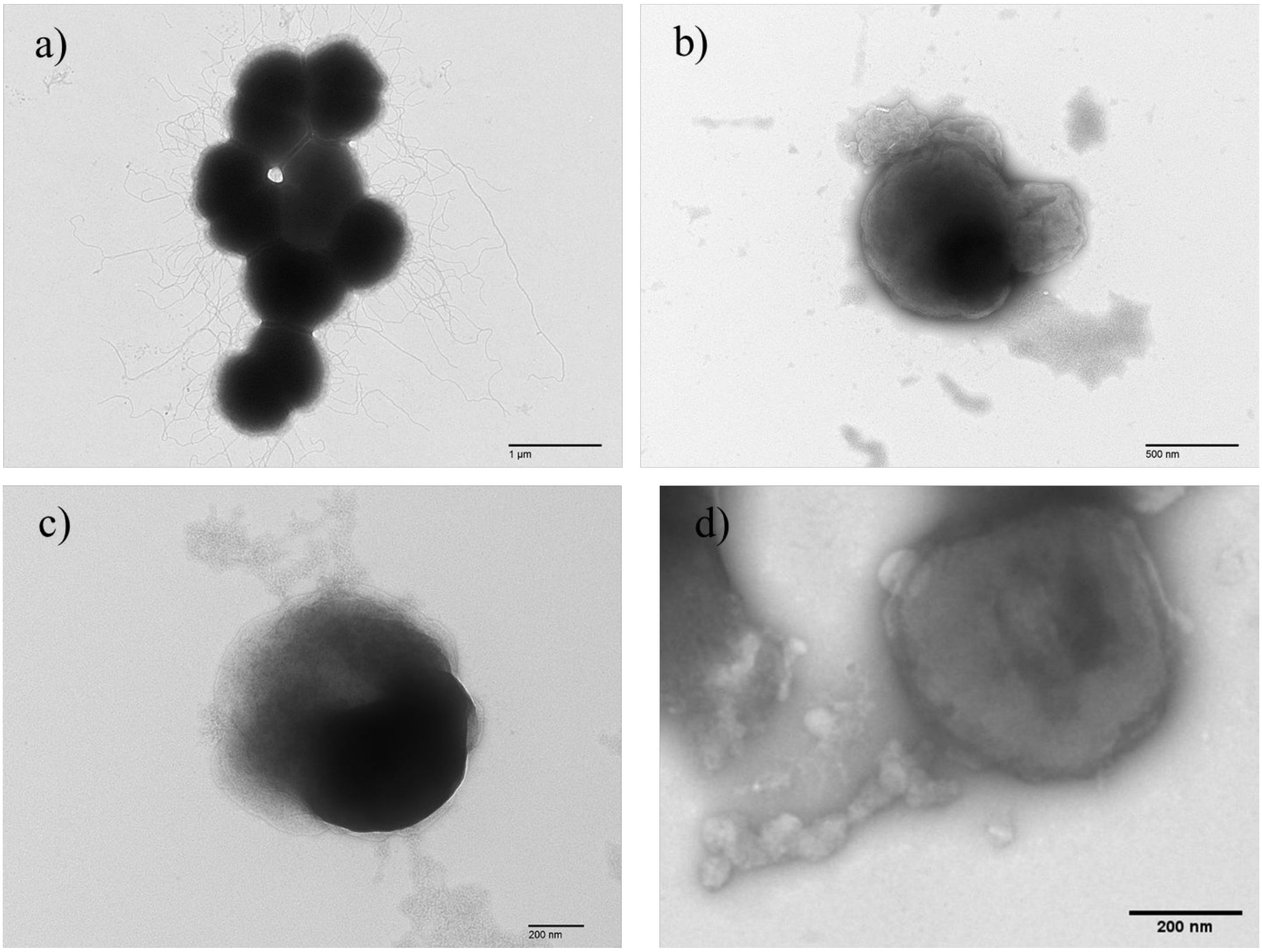

References

1. Sun, D.; Cao, R.; Bi, W.; Weng, J.; Hong, M.; Liang, Y., Syntheses and characterizations of a series of silver-carboxylate polymers. *Inorganica Chimica Acta* **2004,** *357* (4), 991-1001.
2. Gutiérrez, M.; Martín, C.; Souza, B. E.; Van der Auweraer, M.; Hofkens, J.; Tan, J.-C., Highly luminescent silver-based MOFs: Scalable eco-friendly synthesis paving the way for photonics sensors and electroluminescent devices. *Applied Materials Today* **2020,** *21*, 100817.
3. Arenas-Vivo, A.; Celis Arias, V.; Amariei, G.; Rosal, R.; Izquierdo-Barba, I.; Hidalgo, T.; Vallet-Regí, M.; Beltrán, H. I.; Loera-Serna, S.; Horcajada, P., Antiadherent AgBDC Metal-Organic Framework Coating for Escherichia coli Biofilm Inhibition. *Pharmaceutics* **2023,** *15* (1).
4. Akhbari, K.; Morsali, A., Thermal, solution and structural studies of a 3D Ag(I) coordination polymer with various Ag-Ag bonds, [Ag3(μ-Hbtc)(μ-H2btc)]n. *Journal of the Iranian Chemical Society* **2008,** *5* (1), 48-56.
5. Yu, W.; Chen, S.; Zhu, J.; He, Z.; Song, S., A highly dispersed and surface-active Ag-BTC catalyst with state-of-the-art selectivity in CO2 electroreduction towards CO. *Journal of CO2 Utilization* **2023,** *70*, 102457.

6. Seyedpour, S. F.; Dadashi Firouzjaei, M.; Rahimpour, A.; Zolghadr, E.; Arabi Shamsabadi, A.; Das, P.; Akbari Afkhami, F.; Sadrzadeh, M.; Tiraferri, A.; Elliott, M., Toward Sustainable Tackling of Biofouling Implications and Improved Performance of TFC FO Membranes Modified by Ag-MOF Nanorods. *ACS Appl Mater Interfaces* **2020,** *12* (34), 38285-38298.
7. Grancha, T.; Ferrando-Soria, J.; Cano, J.; Amorós, P.; Seoane, B.; Gascon, J.; Bazaga-García, M.; Losilla, E. R.; Cabeza, A.; Armentano, D.; Pardo, E., Insights into the Dynamics of Grotthuss Mechanism in a Proton-Conducting Chiral bioMOF. *Chemistry of Materials* **2016,** *28* (13), 4608-4615.
8. Binaeian, E.; Nabipour, H.; Ahmadi, S.; Rohani, S., The green synthesis and applications of biological metal-organic frameworks for targeted drug delivery and tumor treatments. *J Mater Chem B* **2023,** *11* (48), 11426-11459.
9. Vaidya, L. B.; Nadar, S. S.; Rathod, V. K., Biological metal organic framework (bio-MOF) of glucoamylase with enhanced stability. *Colloids Surf B Biointerfaces* **2020,** *193*, 111052.
10. Huang, Z.-L.; Drillon, M.; Masciocchi, N.; Sironi, A.; Zhao, J.-T.; Rabu, P.; Panissod, P., Ab-Initio XRPD Crystal Structure and Giant Hysteretic Effect (Hc = 5.9 T) of a New Hybrid Terephthalate-Based Cobalt(II) Magnet. *Chemistry of Materials* **2000,** *12* (9), 2805-2812.
11. Ahsan, M. A.; Fernandez-Delgado, O.; Deemer, E.; Wang, H.; El-Gendy, A. A.; Curry, M. L.; Noveron, J. C., Carbonization of Co-BDC MOF results in magnetic C@Co nanoparticles that catalyze the reduction of methyl orange and 4-nitrophenol in water. *Journal of Molecular Liquids* **2019,** *290*, 111059.
12. Uflyand, I. E.; Zhinzhilo, V. A.; Bryantseva, J. D., Synthesis and Study of Sorption, Antioxidant and Antibacterial Properties of MOF based on Cobalt Terephthalate and 1,10-Phenanthroline. *Journal of Inorganic and Organometallic Polymers and Materials* **2021,** *31* (12), 4710-4721.
13. Park, K. S.; Ni, Z.; Côté, A. P.; Choi, J. Y.; Huang, R.; Uribe-Romo, F. J.; Chae, H. K.; O'Keeffe, M.; Yaghi, O. M., Exceptional chemical and thermal stability of zeolitic imidazolate frameworks. *Proc Natl Acad Sci U S A* **2006,** *103* (27), 10186-10191.
14. Schejn, A.; Balan, L.; Falk, V.; Aranda, L.; Medjahdi, G.; Schneider, R., Controlling ZIF-8 nano- and microcrystal formation and reactivity through zinc salt variations. *CrystEngComm* **2014,** *16* (21), 4493-4500.
15. Li, N.; Zhou, L.; Jin, X.; Owens, G.; Chen, Z., Simultaneous removal of tetracycline and oxytetracycline antibiotics from wastewater using a ZIF-8 metal organic-framework. *J Hazard Mater* **2019,** *366*, 563-572.
16. Banerjee, R.; Phan, A.; Wang, B.; Knobler, C.; Furukawa, H.; O'Keeffe, M.; Yaghi, O. M., High-throughput synthesis of zeolitic imidazolate frameworks and application to CO2 capture. *Science* **2008,** *319* (5865), 939-43.
17. Feng, S.; Zhang, X.; Shi, D.; Wang, Z., Zeolitic imidazolate framework-8 (ZIF-8) for drug delivery: A critical review. *Frontiers of Chemical Science and Engineering* **2021,** *15* (2), 221-237.
18. Lebedev, O. I.; Millange, F.; Serre, C.; Van Tendeloo, G.; Férey, G., First Direct Imaging of Giant Pores of the Metal−Organic Framework MIL-101. *Chemistry of Materials* **2005,** *17* (26), 6525-6527.
19. Hajibabaei, M.; Amini, M. M.; Zendehdel, R.; Nasiri, M. J.; Peymani, A., Synthesis, characterization and antibacterial activity of imidazole-functionalized Ag/MIL-101(Cr). *Journal of Porous Materials* **2019**, 1-9.
20. Carson, C. G.; Hardcastle, K.; Schwartz, J.; Liu, X.; Hoffmann, C.; Gerhardt, R. A.; Tannenbaum, R., Synthesis and Structure Characterization of Copper Terephthalate Metal–Organic Frameworks. *European Journal of Inorganic Chemistry* **2009,** *2009* (16), 2338-2343.
21. Nivetha, R.; Sajeev, A.; Mary Paul, A.; Gothandapani, K.; Gnanasekar, S.; Bhardwaj, P.; Jacob, G.; Sellappan, R.; Raghavan, V.; N, K. C.; Pitchaimuthu, S.; Jeong, S. K.; Nirmala Grace,

A., Cu based Metal Organic Framework (Cu-MOF) for electrocatalytic hydrogen evolution reaction. *Materials Research Express* **2020,** *7* (11), 114001.
22. Ghasemzadeh, M. A.; Abdollahi-Basir, M. H.; Mirhosseini-Eshkevari, B., Multi-component synthesis of spiro[diindeno[1,2-b:2′,1′-e]pyridine-11,3′-indoline]-triones using zinc terephthalate metal-organic frameworks. *Green Chemistry Letters and Reviews* **2018,** *11* (1), 47-53.
23. Chui, S. S.; Lo, S. M.; Charmant, J. P.; Orpen, A. G.; Williams, I. D., A chemically functionalizable nanoporous material. *Science* **1999,** *283* (5405), 1148-50.
24. Hirscher, M.; Yartys, V. A.; Baricco, M.; Bellosta von Colbe, J.; Blanchard, D.; Bowman, R. C.; Broom, D. P.; Buckley, C. E.; Chang, F.; Chen, P.; Cho, Y. W.; Crivello, J.-C.; Cuevas, F.; David, W. I. F.; de Jongh, P. E.; Denys, R. V.; Dornheim, M.; Felderhoff, M.; Filinchuk, Y.; Froudakis, G. E.; Grant, D. M.; Gray, E. M.; Hauback, B. C.; He, T.; Humphries, T. D.; Jensen, T. R.; Kim, S.; Kojima, Y.; Latroche, M.; Li, H.-W.; Lototskyy, M. V.; Makepeace, J. W.; Møller, K. T.; Naheed, L.; Ngene, P.; Noréus, D.; Nygård, M. M.; Orimo, S.-i.; Paskevicius, M.; Pasquini, L.; Ravnsbæk, D. B.; Veronica Sofianos, M.; Udovic, T. J.; Vegge, T.; Walker, G. S.; Webb, C. J.; Weidenthaler, C.; Zlotea, C., Materials for hydrogen-based energy storage – past, recent progress and future outlook. *Journal of Alloys and Compounds* **2020,** *827*, 153548.
25. Zhang, X.; Peng, F.; Wang, D., MOFs and MOF-Derived Materials for Antibacterial Application. *J Funct Biomater* **2022,** *13* (4).
26. Whitfield, T. R.; Wang, X.; Liu, L.; Jacobson, A. J., Metal-organic frameworks based on iron oxide octahedral chains connected by benzenedicarboxylate dianions. *Solid State Sciences* **2005,** *7* (9), 1096-1103.
27. Araya, T.; Jia, M.; Yang, J.; Zhao, P.; Cai, K.; Ma, W.; Huang, Y., Resin modified MIL-53 (Fe) MOF for improvement of photocatalytic performance. *Applied Catalysis B: Environmental* **2017,** *203*, 768-777.
28. Sheta, S. M.; Salem, S. R.; El-Sheikh, S. M., A novel Iron(III)-based MOF: Synthesis, characterization, biological, and antimicrobial activity study. *Journal of Materials Research* **2022,** *37* (14), 2356-2367.
29. Horcajada, P.; Surblé, S.; Serre, C.; Hong, D. Y.; Seo, Y. K.; Chang, J. S.; Grenèche, J. M.; Margiolaki, I.; Férey, G., Synthesis and catalytic properties of MIL-100(Fe), an iron(III) carboxylate with large pores. *Chem Commun (Camb)* **2007,** (27), 2820-2.
30. Huang, X.; Yu, S.; Lin, W.; Yao, X.; Zhang, M.; He, Q.; Fu, F.; Zhu, H.; Chen, J., A metal-organic framework MIL-53(Fe) containing sliver ions with antibacterial property. *Journal of Solid State Chemistry* **2021,** *302*, 122442.
31. Eddaoudi, M.; Kim, J.; Rosi, N.; Vodak, D.; Wachter, J.; O'Keeffe, M.; Yaghi, O. M., Systematic design of pore size and functionality in isoreticular MOFs and their application in methane storage. *Science* **2002,** *295* (5554), 469-72.
32. Abdelhameed, R. M.; Darwesh, O. M.; Rocha, J.; Silva, A. M. S., IRMOF-3 Biological Activity Enhancement by Post-Synthetic Modification. *European Journal of Inorganic Chemistry* **2019,** *2019* (9), 1243-1249.
33. Bhardwaj, N.; Pandey, S. K.; Mehta, J.; Bhardwaj, S. K.; Kim, K. H.; Deep, A., Bioactive nano-metal-organic frameworks as antimicrobials against Gram-positive and Gram-negative bacteria. *Toxicol Res (Camb)* **2018,** *7* (5), 931-941.
34. Carton, A.; Mesbah, A.; Mazet, T.; Porcher, F.; François, M., Ab initio crystal structure of nickel(II) hydroxy-terephthalate by synchrotron powder diffraction and magnetic study. *Solid State Sciences* **2007,** *9* (6), 465-471.
35. Abd El Salam, H. M.; Nassar, H. N.; Khidr, A. S. A.; Zaki, T., Antimicrobial Activities of Green Synthesized Ag Nanoparticles @ Ni-MOF Nanosheets. *Journal of Inorganic and Organometallic Polymers and Materials* **2018,** *28* (6), 2791-2798.

36. Chen, W.; Tan, X.; Li, Y.; Zheng, J.; Y, C., Synthesis, crystal structure and magnetic properties of Ni-3(H2O)(6)(TMA)(3-)(TMA)(2)(3-)center dot 2H(2)O with two-dimensional aperture structure. *Chin J Inorg Chem* **2005,** *21*, 1901-1904.
37. Hadi, M.; Mostaanzadeh, H., Sensitive Detection of Histamine at Metal-Organic Framework (Ni-BTC) Crystals and Multi-Walled Carbon Nanotubes Modified Glassy Carbon Electrode. *Russian Journal of Electrochemistry* **2018,** *54* (12), 1045-1052.
38. Sarkar, A.; Adhikary, A.; Mandal, A.; Chakraborty, T.; Das, D., Zn-BTC MOF as an Adsorbent for Iodine Uptake and Organic Dye Degradation. *Crystal Growth & Design* **2020,** *20* (12), 7833-7839.
39. Pang, K.; Figueroa, J. S.; Tonks, I. A.; Sattler, W.; Parkin, G., 2-Mercapto-1-t-butylimidazolyl as a Bridging Ligand: Synthesis and Structural Characterization of Nickel and Palladium Paddlewheel Complexes. *Inorganica Chim Acta* **2009,** *362* (12), 4609-4615.
40. Raju, P.; Ramalingam, T.; Nooruddin, T.; Natarajan, S., In vitro assessment of antimicrobial, antibiofilm and larvicidal activities of bioactive nickel metal organic framework. *Journal of Drug Delivery Science and Technology* **2020,** *56*, 101560.
41. Dan-Hardi, M.; Serre, C.; Frot, T.; Rozes, L.; Maurin, G.; Sanchez, C.; Férey, G., A new photoactive crystalline highly porous titanium(IV) dicarboxylate. *J Am Chem Soc* **2009,** *131* (31), 10857-9.
42. Li, L.; Wang, X.-S.; Liu, T.-F.; Ye, J., Titanium-Based MOF Materials: From Crystal Engineering to Photocatalysis. *Small Methods* **2020,** *4* (12), 2000486.
43. Eddaoudi, M.; Li, H.; Reineke, T.; Fehr, M.; Kelley, D.; Groy, T. L.; Yaghi, O. M., Design and synthesis of metal-carboxylate frameworks with permanent microporosity. *Topics in Catalysis* **1999,** *9* (1), 105-111.
44. Nakhaei, M.; Akhbari, K.; Kalati, M.; Phuruangrat, A., Antibacterial activity of three zinc-terephthalate MOFs and its relation to their structural features. *Inorganica Chimica Acta* **2021,** *522*, 120353.
45. Chen, Y.; Cai, J.; Liu, D.; Liu, S.; Lei, D.; Zheng, L.; Wei, Q.; Gao, M., Zinc-based metal organic framework with antibacterial and anti-inflammatory properties for promoting wound healing. *Regen Biomater* **2022,** *9*, rbac019.
46. Morris, W.; Stevens, C. J.; Taylor, R. E.; Dybowski, C.; Yaghi, O. M.; Garcia-Garibay, M. A., NMR and X-ray Study Revealing the Rigidity of Zeolitic Imidazolate Frameworks. *The Journal of Physical Chemistry C* **2012,** *116* (24), 13307-13312.
